# Long COVID symptom profiles, workforce participation, and working hours among adults in England: a population-based cohort study

**DOI:** 10.1016/j.lanepe.2026.101770

**Published:** 2026-07-23

**Authors:** Chiara Di Gravio, Viveka Guzmán, Shuang Wu, Emily Cooper, Clare Bambra, Nikki Smith, Alex Piper, Matthew Whitaker, Joshua Elliott, Christina J. Atchison, Graham Cooke, Marc Chadeau-Hyam, Paul Elliott, Helen Ward

**Affiliations:** a Department of Epidemiology and Biostatistics, School of Public Health, Imperial College London, UK; b MRC Centre for Environment and Health, Imperial College London, UK; c UK National Institute for Health and Care Research Imperial Biomedical Research Centre, UK; d Patient Experience Research Centre, School of Public Health, Imperial College London, UK; e Population Health Science Institute, Newcastle University, UK; f REACT-LC, Public Advisory Group, School of Public Health, Imperial College London, UK; g Department of Infectious Disease, Imperial College London, UK

**Keywords:** Long COVID, Employment, England, REACT study, Symptom clusters

## Abstract

**Background:**

Long COVID, marked by ongoing multi-systemic symptoms following COVID-19 infection, can impair ability to maintain employment. However, its relationship to workforce retention and working hours remains unclear.

**Methods:**

Long COVID was defined as symptoms lasting ≥12 weeks post-infection. We analysed data from a late-2022 follow-up survey involving 45,864 participants of the Real-time Assessment of Community Transmission (REACT) Study in England (median follow-up: 23 months). Hierarchical clustering identified symptom groups. Multivariable regressions examined associations between Long COVID, being in paid work, and changes in working hours.

**Findings:**

Of 45,864 participants employed at recruitment, 86% (N = 39,341) remained in paid work at follow-up and 11% (N = 4877) changed work hours. Approximately 4% (N = 1967/45,864) had unresolved Long COVID. Compared with participants with no/short (<4 weeks) symptoms, those with unresolved Long COVID had lower odds of being in paid work at follow-up (adjusted odds ratio [aOR]: 0.62, 95% confidence interval [CI]: 0.55, 0.70), and higher odds of changing work hours (aOR: 4.34, 95% CI: 3.88, 4.85). Three clusters were identified: multisystem severe, fatigue-predominant and anosmia-predominant Long COVID. Compared with the fatigue-predominant cluster, participants with multisystem severe Long COVID had lower odds of paid work (aOR: 0.63, 95% CI: 0.47, 0.84) and higher odds of changing work hours (aOR 2.76, 95% CI 2.21, 3.46).

**Interpretation:**

Unresolved Long COVID was associated with worse employment outcomes. Symptom clusters highlighted the importance of considering heterogeneity in Long COVID when assessing workforce impacts and designing public health responses.

**Fundings:**

10.13039/501100000272National Institute for Health and Care Research, 10.13039/100014013UK Research and Innovation.

**Research in context**

**Evidence before this study**

We conducted a search in PubMed for studies published between March 1, 2020, and October 20, 2025. The search was limited to papers written in English. We used the search terms (“long COVID”[Title] OR “Post COVID-19 syndrome”[All Fields]) AND (“employment”[All Fields]). The search returned 88 studies including quantitative analyses, qualitative analyses and mixed-methods research. Overall, Long COVID has been associated with reduced productivity, increased sick leave, diminishing working hours and workforce exit across multiple countries. However, the extent to which specific Long COVID symptom profiles are linked to employment changes remains poorly understood.

**Added value of this study**

We leverage baseline and follow-up data collected as part of the Real Time Assessment of Community Transmission Study: a large community-based study across England set up in early 2020 to track prevalence of COVID-19 infections. The data includes a heterogeneous group of people, including individuals who have never had a COVID-19 infection at the time of survey. Using a data-driven approach, we derived Long COVID symptoms profiles to account for potential heterogeneity in the relation between Long COVID and employment.

**Implications of all the available evidence**

Our findings suggest potential long-term disruptions to employment for people with Long COVID and shows how different Long COVID symptoms profiles are related to employment outcomes. The results highlight the importance of accounting for heterogeneity in Long COVID when assessing workforce implications and designing public health strategies.

## Introduction

The COVID-19 pandemic has had widespread health, social and economic consequences worldwide.^1^^,^^2^ While much attention has focused on the acute phase, evidence shows that a subset of individuals experience persistent, multisystemic symptoms following SARS-CoV-2 infection—a condition now recognised as Long COVID.^3^ According to the 2024 National Academies of Sciences, Engineering, and Medicine definition, these symptoms must persist for at least three months in a continuous, relapsing and remitting, or progressive disease state affecting one or more organ systems.^3^ Such symptoms can limit daily functioning, including the capacity to engage in paid work.

Prevalence estimates for Long COVID vary by case definition and study design, complicating efforts to quantify its population-level impact.^4^ The World Health Organization (WHO) estimates that, worldwide, 10–20% of people with COVID-19 experience symptoms beyond the acute phase.^5^ By March 2024, 3.3% of the population in England and Scotland reported COVID-19 symptoms lasting more than four weeks, with higher prevalence among adults aged 45–64, and those not working or seeking work (9.1%).^6^

Since 2020, economic inactivity, defined as being out of employment and not seeking or being able to start working, has increased in the context of long-term sickness and disability, posing substantial challenges for labour markets and national economies.^7^ Evidence from multiple settings suggests that Long COVID contributes to this trend through reduced productivity, increased sick leave, diminished working hours, and workforce exit.^7^, ^8^, ^9^, ^10^, ^11^ However, treating Long COVID as a single, binary condition may hide important heterogeneity in symptoms presentation. Previous clustering analyses have characterised distinct Long COVID phenotypes, ranging from mild symptoms burden to higher severities associated with diverse functional impairments.^12^, ^13^, ^14^ Such heterogeneity may influence individuals’ capacity to engage in paid employment, yet the extent to which specific symptom profiles are associated with employment changes remains poorly understood.

Using data from the Real-time Assessment of Community Transmission (REACT) Study, we examine factors associated with employment changes between 2020 and 2022. Particularly, we aim to quantify the association between Long COVID and its symptom profiles with remaining in paid work and changes in paid work hours. We hypothesise that individuals with unresolved Long COVID will be less likely to remain in paid employment, and more likely to change their hours of work.

## Methods

### The REACT study

**Fig. 1:**
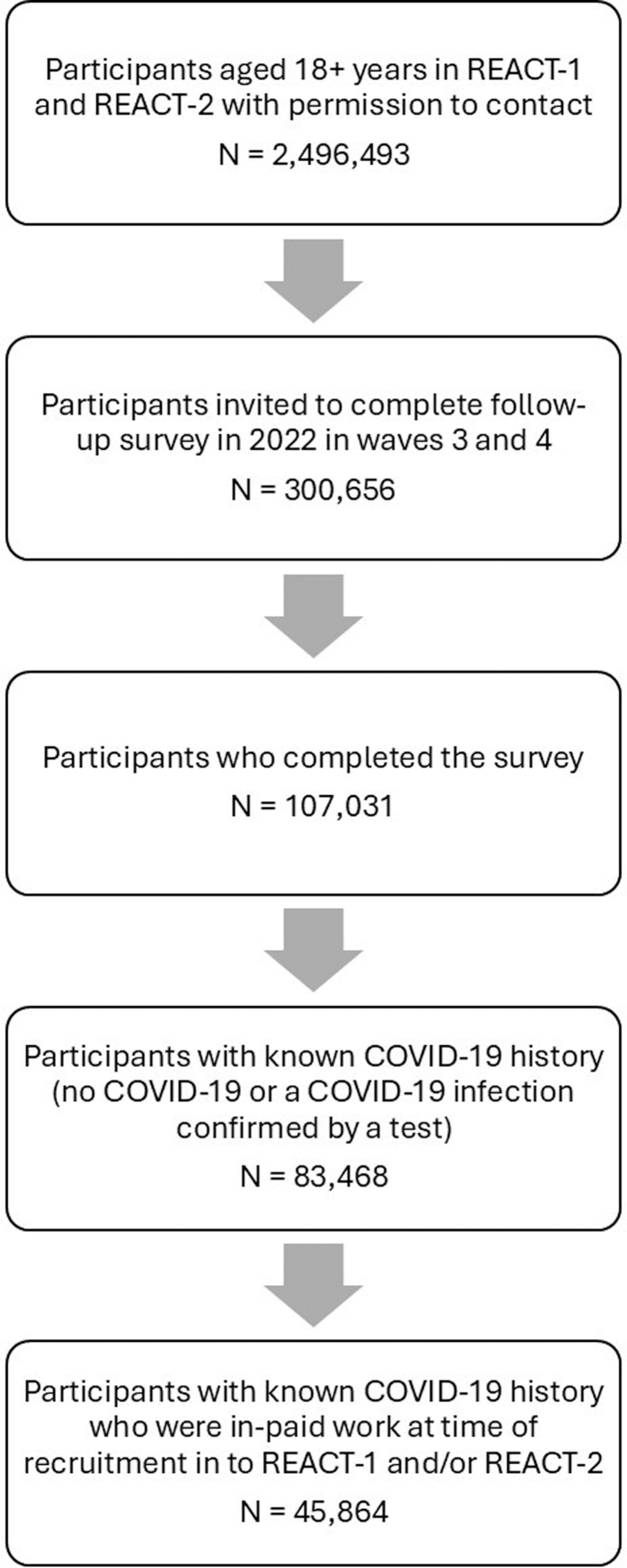
Flowchart of the study sample.

The REACT study, launched in April 2020 to track SARS-CoV-2 infections in England, comprised two components recording the prevalence of SARS-CoV-2 infections (REACT-1: 19 rounds, May 2020–March 2022) and the prevalence of IgG antibody to the SARS-CoV-2 spike protein (REACT-2: 6 rounds. June 2020–May 2021). Information on data collection is available elsewhere.^15^^,^^16^ Briefly, participants were randomly sampled from the National Health Service patient list and information on SARS-CoV-2 infections, comorbidities, socio-demographics, and employment was collected via home testing and online questionnaires. Between August 2022 and December 2022, a non-random sub-sample of consenting participants was invited to complete a follow-up survey as part of the REACT-Long COVID (REACT-LC) study (Supplementary Material SM1 for additional details).^17^ This paper includes the 107,029 participants who completed the follow-up survey between October and December 2022. Among these, we considered the 45,864 (43%) participants who were in paid employment (working full-time, part-time or self-employed) at first enrolment in REACT, and either reported no COVID-19 infection or at least one infection confirmed by a positive swab/polymerase chain reaction (PCR)/antigen/lateral flow swab test (Fig. 1). The REACT-LC study holds ethical approval from South-Central Berkshire B Research Ethics Committee (IRAS IDs: 298404, 259978, 283787, 298724). All participants provided written informed consent.

### Definition of employment outcomes

We defined two outcomes using participants’ self-reported information on employment nature, and changes in paid work hours. First, we classified participants as being “in paid work” if they were in any full-time job, part-time job or they were self-employed at time of the 2022 follow-up survey. Then, we used the responses to the question: “Since March 2020, has your physical or mental health affected the number of hours of paid work that you can do?” to describe changes in hours of paid work (Supplementary Material SM2).

### Definition of Long COVID status

Participants were classified into five groups: those with no history of COVID-19 (no COVID-19); those with a test-confirmed infection who were asymptomatic or whose symptoms resolved within 4 weeks (asymptomatic or resolved COVID-19 <4 weeks); those with a test-confirmed infection whose symptoms resolved between 4 and 11 weeks (resolved COVID-19, 4–11 weeks); those with a test-confirmed infection and symptoms lasting ≥12 weeks but now resolved (resolved Long COVID); and those with symptoms lasting ≥12 weeks and still ongoing (unresolved Long COVID).

For participants reporting multiple COVID-19 infections, classification was based on the longest test-confirmed episode, using information from first and most recent infections, as these were the episodes for which data were collected in the follow-up survey. Because participants enrolled earlier in the pandemic might not have had access to testing, we created a secondary exposure that applied the same classification but also included self-reported suspected infections. The research questions and workflow used to create the exposure are summarised in the Supplementary Material (SM3 and Supplementary Figure S1).

### Statistical analysis

We used multivariable logistic regression models to examine associations between Long COVID status and employment outcomes. Models were adjusted for a pre-specified set of covariates selected based on the directed acyclic graph in Supplementary Figure S2. First, we fit minimally-adjusted models that included employment nature (full-time, part-time, self-employed) and type (business and services, child-related, health care, care home, logistic/security, other essential worker, work at home/not public facing) at time of recruitment in REACT, gender, age, education level, number of previous comorbidities, ethnicity, and taking precautions due to concern about severe COVID-19 illness (yes/no) at time of recruitment. Afterwards, we fit fully-adjusted models that further include financial impact of two weeks off-work to illness and the 2019 Index of Multiple Deprivation (IMD). IMD is a measure of relative deprivation for small geographical area across England (approximately 1500 residents per area) calculated based on seven domains (income, employment, education, health, crime, barriers to housing and services, and living environment). Participants were allocated to quintiles of IMD based on their residential postcodes.^18^ Survey questions used to define all variables are listed in the Supplementary Material. For analysis on whether participants were still in paid work, we further adjusted the models for time between recruitment in REACT and follow-up, as participants entered each survey at different times (REACT study: April 30, 2020–March 17, 2022; follow-up survey: October 24, 2022–December 29, 2022). We modelled age using restricted cubic splines with knots placed at the 33rd and 67th percentiles.

We assessed effect modification by gender and socio-economic status by fitting the fully-adjusted models with the additional inclusion of interaction terms between Long COVID status, gender and IMD.

To characterise patterns of persistent symptoms among participants with resolved and unresolved Long COVID, we applied hierarchical clustering to the 29 surveyed symptoms (a complete list of symptoms is in the Supplementary Material SM4). Hierarchical clustering starts by treating each participant as its own cluster and iteratively groups them based on similarity (quantified by the Euclidean distance) in symptoms profiles. To minimise within-cluster variance and produce easy-to-interpret groups, we applied Ward's distance as the linkage criterion. We evaluated two to ten clusters selecting the optimal number based on cluster stability.^19^

Finally, we examined associations between symptom clusters and employment outcomes using additional multivariable logistic regression models adjusted for the clusters and the same set of covariates described above. In all analyses, we imputed missing data using multiple imputation with predictive mean matching and ten imputed datasets. Estimated coefficients and standard errors were combined using Rubin's rule.^20^ Details on the multiple imputation algorithm are in the Supplementary Material (SM6). Analyses were conducted in R v4.2.0.^19^^,^^21^^,^^22^

#### Sensitivity analyses

We repeated all analyses using Long COVID status derived from both self-reported suspected cases and test-confirmed cases.

Because COVID-19 infections in the REACT study could occur either before or after recruitment, baseline employment might be both a cause and an effect of Long COVID. Moreover, as baseline employment is related to follow-up employment, to assess the robustness of our results to potential biases, we repeated all analyses considering 1) all REACT 2022 follow-up participants regardless of their baseline employment status, and 2) participants who were in-paid employment at baseline and did not have a COVID-19 infection before recruitment in the REACT study (i.e., those who either never had COVID-19 or developed their first infection after recruitment).

Finally, to assess robustness of symptom clusters to the choice of algorithm, we applied Partitioning Around Medoids (PAM) to the same set of participants and symptoms (Supplementary Material SM8).^23^

### Role of funding source

The funders had no role in study design, data collection and analysis, results interpretation, preparation of the manuscript or decision to publish.

## Results

Participants who completed the follow-up survey were similar to those invited in terms of health, ethnicity and socio-economic status, although responders were more often older and retired. Among the 45,864 participants included in the study the majority (28,862/45,864 = 63%) were full-time workers at recruitment in REACT, followed by part-time workers (10,129/45,864 = 22%) and self-employed (6873/45,864 = 15%). Over half of the participants (24,593/45,864 = 55%) were working at home or in a non-public facing role. At follow-up, 86% (39,341/45,864) were still in paid work while 11% (4877/45,864) reported having changed their hours of paid work (Table 1). Compared with the general adult population, follow-up participants were more likely to be female, older, of white ethnicity and from the less deprived areas (Supplementary Table S1).

**Table 1:** Characteristics of participants.^a^

	Participants invited to complete the REACT follow-up survey in Oct–Dec 2022 (N = 300,656)	Participants who completed the REACT follow-up survey in Oct–Dec 2022 (N = 107,029)	Participants with known COVID-19 status who were in paid work at time of recruitment in REACT (N = 45,864)
Employment nature at recruitment in REACT			
Full-time	120,532 (41.3%)	37,311 (35.8%)	28,862 (63.0%)
Part-time	36,987 (12.7%)	12,944 (12.4%)	10,129 (22.0%)
Retired	74,525 (25.5%)	35,545 (34.1%)	–
Self-employed	28,353 (9.7%)	9140 (8.8%)	6873 (15.0%)
Sick/disabled	5549 (1.9%)	1818 (1.7%)	–
Student	22,740 (3.0%)	1901 (1.8%)	–
Not working	17,355 (5.9%)	5539 (5.3%)	–
Missing	8699	2831	–
Employment type at recruitment in REACT^b^			
Business and service	11,092 (3.9%)	3151 (3.1%)	2330 (5.2%)
Care home	2178 (0.8%)	702 (0.7%)	543 (1.2%)
Child-related	14,950 (5.2%)	5440 (5.3%)	4293 (9.6%)
Health care worker	16,897 (5.9%)	6412 (6.2%)	5159 (12.0%)
Logistic and security	5590 (2.0%)	1724 (1.7%)	1284 (2.9%)
Not in full-time, part-time nor self-employed	106,085 (37.1%)	44,803 (43.6%)	–
Other essential worker	29,861 (10.4%)	8627 (8.4%)	6532 (15.0%)
Work at home/not public facing	99,584 (34.8%)	31,863 (31.0%)	24,593 (55.0%)
Missing	14,599	4307	1130
Gender			
Male	129,852 (43.2%)	44,188 (41.3%)	18,444 (40.0%)
Female	170,800 (56.8%)	62,839 (58.7%)	27,419 (60.0%)
Missing	4	2	1
Age (years)	55 (41, 66)	59 (47, 68)	52 (42, 60)
Ethnicity			
Asian	10,269 (3.4%)	2900 (2.7%)	1375 (3.0%)
Black	2778 (0.9%)	815 (0.8%)	411 (0.9%)
Mixed	3937 (1.3%)	1223 (1.2%)	650 (1.4%)
Other	3652 (1.2%)	1219 (1.2%)	529 (1.2%)
White	277,886 (93.1%)	100,072 (94.2%)	42,705 (94.0%)
Missing	2134	800	194
Education level			
Degree level or higher	117,010 (40.4%)	46,665 (44.8%)	23,284 (51.5%)
Other higher qualification below degree level	30,838 (10.5%)	12,015 (11.6%)	4663 (10.3%)
A-levels, NVQ levels 3 and equivalents	48,811 (16.9%)	15,380 (14.8%)	7364 (16.3%)
GCSE/O level	41,460 (14.3%)	13,488 (12.9%)	5356 (11.9%)
Qualification at level 1 and below	14,481 (5.0%)	4542 (4.4%)	1708 (3.8%)
Other qualification	19,100 (66%)	7086 (11.5%)	1900 (4.2%)
No qualification	18,139 (6.3%)	4995 (4.8%)	899 (2.0%)
Prefer not to say/Missing	11,272	2858	690
IMD quintile			
Q1—most deprived	26,980 (9.8%)	8315 (8.4%)	3084 (9.0%)
Q2	43,561 (15.9%)	14,936 (15.1%)	6737 (16.0%)
Q3	58,878 (21.5%)	21,255 (21.5%)	9257 (22.0%)
Q4	68,177 (24.8%)	24,996 (25.3%)	10,611 (25.0%)
Q5—least deprived	76,846 (28.0%)	29,388 (29.7%)	12,055 (28.0%)
Missing	26,214	8139	3400
If you were off work for two weeks due to illness how serious would the financial impact be on your household?			
Not serious at all	28,726 (41.8%)	12,235 (45.6%)	9910 (47.0%)
Not very serious	21,291 (31.0%)	8249 (30.7%)	6359 (30.0%)
Fairly serious	12,620 (18.4%)	4336 (16.2%)	3310 (16.0%)
Very serious	6132 (8.9%)	2011 (7.5%)	1441 (7.0%)
Missing	231,887	70,715	24,844
Are you taking specific precautions because you are concerned that you will become severely ill with COVID-19?			
Yes	38,004 (13.0%)	14,855 (14.2%)	3883 (8.7%)
Missing	9294	2618	1130
Number of comorbidities^c^			
0	163,855 (54.5%)	57,193 (53.4%)	37,235 (59.0%)
1	81,385 (27.1%)	29,944 (28.0%)	11,918 (26.0%)
2+	55,416 (18.4%)	18,892 (18.6%)	6711 (15.0%)

a For categorical variables we report frequency and percentage. Percentages are calculated from those who answered the question of interest in the follow-up survey. For continuous variables we report median and interquartile range. IMD, index of multiple deprivation (measure of relative deprivation at small local area level across England).

b Business and service includes individuals working in food retail, hospitality and personal care. Child-related includes individuals working in schools, nurseries, childcare centres or providing childcare services. Logistic and security includes individuals working in home deliveries, public transit, police, prison, fire and rescue, coastguard, and armed forces. Health care workers and care home workers include both workers with and without direct contact with patients/clients. Other essential workers/public facing roles include all other essential workers that were not included in the other groups based on Government guidelines.^5^

c Number of self-reported health conditions at time of recruitment in the REACT study (organ transplant recipient; diabetes heart disease or heart problems; hypertension; stroke; kidney disease; liver disease; anaemia; asthma; lung conditions such as COPD, bronchitis or emphysema; cancer; conditions affecting the brain and nerves such as dementia, Parkinson's disease and multiple sclerosis; a weakened immune system/reduced ability to deal with an infection as a result of disease or treatment; depression; anxiety; and psychiatric disorder).

Median time between recruitment in REACT and recruitment in the follow-up was 23 months (interquartile range [IQR]: 15, 27 months). Overall, participants’ nature of employment did not change substantially with most participants in full-time, part-time or self-employment at baseline remaining in the same category at follow-up (23,765/27,646 = 85.9%, 6173/9604 = 64.3% and 4946/6507 = 76.0%, respectively). Details are presented in Supplementary Figure S3.

Of participants employed at recruitment in REACT, 74.1% (35,245/45,864) had symptomatic COVID-19 while 3.4% (1547/45,864) reported an asymptomatic infection. Approximately 2.3% (1053/45,864) and 4.3% (1967/45,864) of participants had resolved and unresolved Long COVID, respectively (Table 2). Compared with those without COVID-19, participants with Long COVID were younger, female, and more likely to be working in public facing roles. Over 80% (2640/3020) of participants with Long COVID had moderate or severe symptoms during their acute COVID-19 infection (Supplementary Table S2). Among full-time workers at recruitment in REACT, 1.9% (22/1141) of participants with unresolved Long COVID were not working at follow-up due to sickness. The percentage reduced to 0.43% (26/6113) for full-time workers at recruitment without COVID-19 (Supplementary Figure S4).

**Table 2:** Frequency and prevalence of Long COVID status among participants included in the primary analysis.

Long COVID status	Participants with known COVID-19 status who were in paid work at time of recruitment in REACT (N = 45,864)
No COVID-19	10,619 (23.2%)
Asymptomatic or resolved short COVID-19 less than 4 weeks	30,008 (65.4%)
*Of which…*	
*Without symptoms*	*1547*
*With symptoms*	*28,461*
Resolved COVID-19 (4–11 weeks)	2217 (4.8%)
Resolved Long COVID	1053 (2.3%)
Unresolved Long COVID	1967 (4.3%)

Participants who were in the ‘Asymptomatic or resolved short COVID-19 less than 4 weeks’ category were further divided based on whether they reported any symptom.

In fully adjusted models, compared with participants who were asymptomatic or had symptoms lasting <4 weeks, participants with unresolved Long COVID had 38% lower odds of being in paid work at follow-up (adjusted odds ratio [aOR]: 0.62, 95% CI: 0.55, 0.70) and four times higher odds of changing their work hours (aOR: 4.34, 95% CI: 3.88, 4.85). The odds of changing work hours decreased with shorter symptoms duration (Table 3). Compared to full-time workers, part-time and self-employed participants had 45% (aOR: 0.55, 95% CI: 0.51, 0.59) and 15% (aOR: 0.85, 95% CI: 0.7,8 0.93) lower odds of being in paid work at follow-up, while they had higher odds of changing their work hours (aOR: 1.40, 95% CI: 1.28, 1.53 for part-time workers and aOR: 2.03, 95% CI: 1.86, 2.23 for self-employed workers). Finally, compared to those working from home or in non-public facing roles, participants in any public facing roles had similar odds of being in paid work at follow-up, but higher odds of changing their work hours (Supplementary Figures S4 and S5). Minimally-adjusted models produced similar estimates (Supplementary Figures S6 and S7). Associations between Long COVID status and being in paid work at follow-up were similar across gender or IMD (Supplementary Tables S3 and S4).

**Table 3:** Associations between Long COVID status and symptom clusters with being in paid work and changes in hours of work at follow-up.

	In paid work at time of recruitment in the follow-up survey	Changes in hours of paid work
	aOR (95% CI)	aOR (95% CI)
Model #1 exposure: Long COVID status		
No COVID	0.98 (0.92, 1.05)	0.97 (0.90, 1.05)
Asymptomatic or resolved short COVID-19 <4 weeks	Reference	Reference
Resolved COVID-19 (4–11 weeks)	0.95 (0.83, 1.08)	1.91 (1.69, 2.16)
Resolved Long COVID	0.80 (0.67, 0.96)	1.91 (1.60, 2.28)
Unresolved Long COVID	0.62 (0.55, 0.70)	4.34 (3.88, 4.85)
Model #2 exposure: Long COVID symptoms clusters		
C1: Fatigue-predominant Long COVID	Reference	Reference
C2: Long COVID characterised by loss/change of smell and/or taste	1.22 (0.85, 1.71)	0.64 (0.49, 0.85)
C3: Multisystem severe Long COVID	0.63 (0.47, 0.84)	2.76 (2.21, 3.46)

Adjusted odds ratio (aOR) and 95% confidence intervals (CI). Models were adjusted for nature of employment at time of recruitment in the REACT study, type of employment at time of recruitment in the REACT study, gender, age, education level, previous comorbidities, IMD (index of multiple deprivation) quintiles, ethnicity, taking precautions at time of recruitment in the REACT study due to worries about becoming ill (yes/no), and financial impact in case of having to take two weeks off work due to illness. When the outcome was whether a participant was in paid work at time in the follow-up survey, we additionally adjusted for months between recruitment in the REACT study and recruitment in the follow-up survey.

Results are presented for the fully-adjusted model.

Overall, being in paid work at follow-up was mainly explained by age and nature of employment at recruitment, whereas changes in work hours were mainly explained by previous comorbidities and Long COVID status (Supplementary Figure S9).

### Symptoms clusters

Among the 2208 participants with Long COVID and symptoms data, the most common symptoms lasting ≥12 weeks were mild fatigue (1430/2208 = 65%), brain fog (1205/2208 = 55%) and shortness of breath (1089/2208 = 49%) (Supplementary Figure S10).

**Fig. 2:**
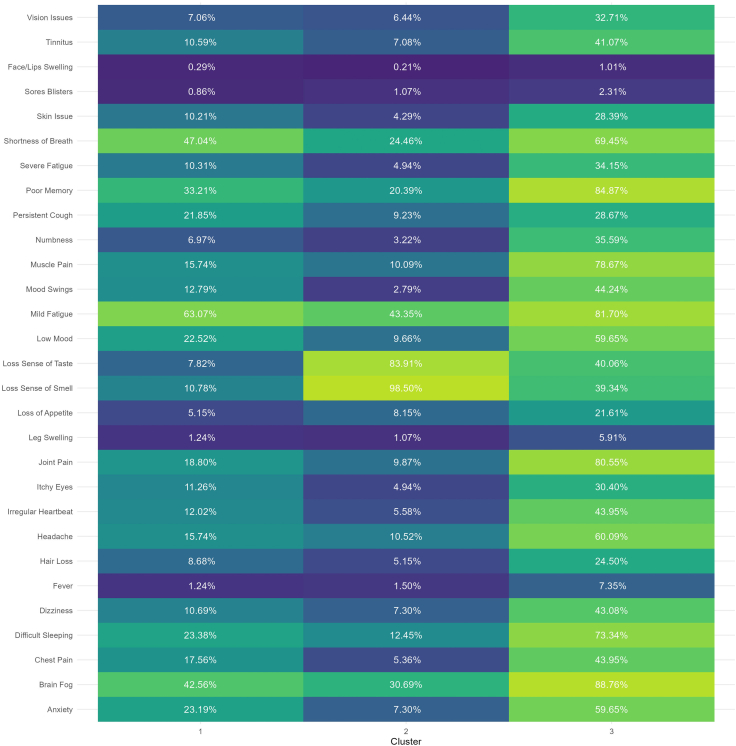
Clusters of persistent symptoms lasting at least 12 weeks among participants with resolved and unresolved Long COVID. Clusters were derived using hierarchical clustering. Cluster 1 (N = 1048) describes a fatigue-predominant Long COVID. Cluster 2 (N = 466) describes Long COVID characterised mainly by loss/change of smell and taste. Cluster 3 (N = 694) describes multisystem severe Long COVID.

Cluster analysis identified three clusters based on visual inspection of the dendrogram and the consensus score plot (Supplementary Figures S11 and S12): a fatigue-predominant Long COVID cluster (C1), an anosmia-dominant Long COVID cluster (C2) and a multisystem severe Long COVID cluster (C3). Most participants were in C1 (1048/2208 = 47%) followed by C3 (694/2208 = 32%) and C2 (466/2208 = 21%). Median number of symptoms for participants in C1, C2 and C3 was 4 (IQR: 2, 7), 4 (IQR: 2, 6) and 12 (IQR: 10, 15), respectively. Fig. 2 shows symptoms prevalence across the three clusters and illustrate how the clusters primarily represent different levels of Long COVID severity, with C3 being the most severe profile, followed by C1 and C2.

Participants in C1, C2 and C3 had similar employment at recruitment, but those in C3 reported more severe COVID-19 symptoms during the acute phase of their infection. At follow-up, most participants remained in paid work (926/1048 = 88.4% in C1, 417/466 = 89.4% in C2 and 566/694 = 81.6% in C3), yet changes in hours of paid work were more common in C3 (282/1048 = 28.6%, 86/466 = 19.6% and 361/694 = 57.1% respectively; Supplementary Table S5). Compared with C1 participants, C3 participants had worse employment outcomes, with 39% lower (aOR: 0.63, 95% CI: 0.47, 0.84) odds of being in paid work at follow-up, and more than twice the odds of changing work hours (aOR; 2.76, 95% CI: 2.21, 3.46). Participants in C3 had lower odds of changing their hours of work compared to those in C1 (aOR: 0.64, 95% CI: 0.49, 0.85; Table 3).

The consensus score plot indicated that a two-cluster solution was similarly stable (Supplementary Figure S12). For completeness, we evaluated this solution (Supplementary Tables S6 and S7, Supplementary Figure S13). Results were consistent with the three-cluster model with clusters mainly reflected different Long COVID severity, and with participants in the most severe cluster having worse employment outcomes (Supplementary Table S7).

### Sensitivity analyses

Extending the Long COVID definition to suspected infections increased the sample to 49,273 participants, of whom 3.1% (2341/49,273) and 4.8% (1521/49,273) had resolved and unresolved Long COVID, respectively (Supplementary Table S8). Compared with participants who were asymptomatic or had symptoms lasting <4 weeks, participants with unresolved Long COVID had 35% lower odds of being in paid work at time of follow-up (aOR: 0.65, 95% CI: 0.58, 0.73) and over four times higher odds of having had to change their work hours (aOR: 4.50, 95% CI: 4.05, 5.01) (Supplementary Table S9).

Sensitivity analyses using different samples—one including all follow-up participants regardless of their baseline employment status, and another restricted to those in paid work at baseline who had not previously had a COVID-19 infection (see Supplementary Table S10 for samples composition—produced results consistent with the primary analysis. In both cases, participants with unresolved Long COVID had worse employment outcomes than those who were asymptomatic or had symptoms lasting <4 weeks, with odds ratios of similar magnitude (Supplementary Tables S11 and S12). Cluster analyses consistently identified the same three clusters reflecting different levels of Long COVID severity (Supplementary Figures S15 and S16, Supplementary Tables S11 and S12).

Clustering using the PAM algorithm resulted in groups that closely matched those identified with hierarchical clustering (Supplementary Figure S17). When comparing the three-cluster solution from hierarchical clustering with the PAM solution, 83% (867/1048), 94% (437/466) and 80% (555/694) of individuals in the were assigned to the same cluster by both algorithms. Agreement percentages were lower when comparing the two-cluster solution from hierarchical clustering with the three-cluster solution from the PAM algorithm (Supplementary Figure S18). As previously observed, participants in the most severe cluster had worse employment outcomes (Supplementary Table S18).

## Discussion

In this study of over 40,000 adults employed at recruitment into REACT, we observed Long COVID in 6.6% (3020/45,864) of participants. Prevalence was higher among participants working in public-facing roles likely due to greater exposure during periods when protective measures and vaccination coverage were limited. By the end of 2022, 14% (6523/45,864) of participants were no longer in paid employment, and 11% (4877/45,864) had changed their work hours due to ill-health. Employment outcomes were worse for people with unresolved Long COVID than for those whose an illness lasting <4 weeks. The findings align with smaller international studies: for example, an Italian study reported that 13.7% participants with previous COVID-19 infection changed the nature of their employment within a year from their infection.^24^ An earlier study recruiting participants through online COVID-19 support groups reported that 45% of participants reduced their work hours, while 22% stopped working, likely reflecting a more vulnerable sample.^25^ In contrast, the REACT study included participants with no infections as well as those with severe, mild or asymptomatic COVID-19.

Consistent with previous evidence showing the differential impact of COVID-19 on self-employed compared to full-time workers,^26^ we found that part-time and self-employed participants were more vulnerable to employment changes than those working full-time. These findings complement qualitative work from the REACT study which highlighted symptoms severity, job responsibilities, occupational support and social networks as factors for changes in employment among people with Long COVID.^27^

We identified three clusters reflecting different levels of Long COVID severity: a multisystem severe cluster, a fatigue-predominant cluster, and an anosmia-dominant cluster. Studies across Europe and the US identified similar patterns that ultimately described Long COVID severity.^12^, ^13^, ^14^^,^^28^ Although the multi-systemic nature of Long COVID and the differential impact on people's lives is increasingly recognised,^29^ research linking symptom clusters to employment outcomes remains limited. Existing studies suggest that individuals in the most severe Long COVID cluster have reduce ability to work and experience longer absences from employment.^12^^,^^13^ In this context, our finding that participants in the anosmia-dominant cluster had better employment outcomes compared to the fatigue-predominant cluster indicates that broader symptoms profiles, rather than isolated symptoms are more relevant for understanding functional limitations in relationship to employment.

REACT is a large, community-based study that initially recruited through a random sample of the general population. Unlike studies recruiting through Long COVID groups, clinical services or hospital-based cohorts, REACT provides a wider range of health experiences by including people without COVID-19 and those with short-lasting infections. Our definition of Long COVID was based on self-reported symptoms duration rather than self-identification with Long COVID, allowing inclusions of individuals who may have limited access to services and less awareness of the term. Results were similar regardless of whether we consider test-confirmed infections only or included test-confirmed and suspected infections.

This study has several limitations. First, we included only participants who were in paid employment at time of recruitment. Because people were recruited between 2020 and early 2022, it is possible that participants had already left the workforce due to the pandemic and/or other health issues (i.e., healthy worker bias) by the time they entered the study, and/or participants had left work before developing Long COVID causing misattribution of directionality. As a COVID-19 infection might have occurred after recruitment in the REACT study, and baseline and follow-up employment are related, restricting analyses to participants employed at recruitment could introduce collider bias. Nonetheless, results from sensitivity analyses using different samples (one including all participants regardless of baseline employment, and one restricted to those employed at baseline who had not previously had a COVID-19 infection) produced results of similar magnitude to those in the main analysis.

Second, because the analysis is based on changes in work hours, it is not possible to distinguish between increases or decreases in work hours. Nonetheless, any deviation from an individual's usual paid working hours is likely to indicate a disruption in work functioning (e.g., diminished capacity to meet work demands, needing to work longer to accomplish the same task) which might impact the mental and physical health of participants with persistent symptoms. While paid hours might have different financial implications for self-employed versus salaried individuals, our qualitative work, suggests that changes in paid hours remain informative across employment types.^27^

Because duration of symptoms was self-reported there is potential for recall bias as reporting could be related to the time since a participant's infection, the knowledge of their condition, and their health behaviour. Moreover, as in any health study, it is possible that participants without Long COVID dropped out from the study. Finally, we do not have data on factors that might impact employment during the pandemic (e.g., whether participants were part of the UK government furlough scheme), nor we have information on 1) pre-pandemic employment 2) potential relapses over time and 3) persistent gastro-intestinal symptoms, which have been previously related to Long COVID experiences.^25^

Unresolved Long COVID, being self-employed, working part-time, and severe symptoms profiles were associated with worse employment outcomes including lower workforce retention, and changes in work hours. These results suggest potential long-term disruptions to employment in people with Long COVID contributing to productivity losses and workforce changes. At the individual level, changes in work hours and nature of employment could have an impact on both financial security and overall wellbeing. Our results suggest the need to account for heterogeneity in Long COVID when assessing workforce impacts and designing public health strategies.

## Contributors

CDG, VG, SW, HW, CJA conceptualised the study. CDG, HW were involved in methodology. CDG, MW and JE have current access to the data. CDG, CJA and SW had accessed and verified a previous version of the data. CDG, SW curated and analysed the data with additional inputs from VG, HW, MW and JE. CDG, VG, HW wrote the original draft. CDG, VG, SW, EC, NS, AP, CB, CJA, GC, MC, PE, HW. JE, MW contributed to reviewing and editing the manuscript. CDG and VG administered the project. GC, MC, PE, HW acquired fundings. All authors have approved the final manuscript. CDG, VG, HW and PE were responsible for the decision to submit the manuscript.

## Data sharing statement

The data that support the findings of this study are not publicly available. Deidentified data can be made available upon reasonable request and in line with the consent agreed with participants, by submitting a methodologically sound research proposal to react.lc.study@imperial.ac.uk.

## Declaration of interests

PE, HW, CDG and VG declare that the work on this manuscript was funded by grants to Imperial College London from the National Institute for Health & Social Care Research and the UK Research and Innovation Research. HW received travel costs and speakers’ fees from BioNTech. All other authors declare no competing interests.
