# Supplementary material for: Long COVID symptom profiles, workforce participation, and working hours among adults in England: a population-based cohort study
Source: Lancet Reg Health Eur. 2026 Jul 23;68:101770. doi: 10.1016/j.lanepe.2026.101770 (PMC13425795; doi:10.1016/j.lanepe.2026.101770)
Supplement: Supplementary Material [file mmc1.docx]

Supplementary Material for “**Long COVID symptom profiles, workforce participation, and working hours among adults in England: a population-based cohort study”**

**METHODS**

[**SM1.** REACT Sampling Design 4](#_Toc228293061)

[**SM2.** Survey Questions Used to Derive the Outcomes 4](#_Toc228293062)

[**SM3.** Survey Questions Used to Derive the Long COVID Exposure 5](#_Toc228293063)

[**SM4.** Survey Questions Used to Derive Symptoms’ Clusters 7](#_Toc228293064)

[**SM5.** Survey Questions Used to Derive Models’ Covariates 7](#_Toc228293065)

[**SM6.** Multiple Imputation 10](#_Toc228293066)

[**SM7.** Cluster Stability 11](#_Toc228293067)

[**SM8.** Partition Around Medoid (PAM) Algorithm 11](#_Toc228293068)

**FIGURES**

[**Supplementary Figure 1**. Pipeline for the computation of the Long COVID status exposure 12](#_Toc232666947)

[**Supplementary Figure 2.** Directed Acyclic Graph (DAG) showing the variables considered when modelling the relationship between Long COVID status and employment outcomes 13](#_Toc232666948)

[**Supplementary Figure 3.** Comparison of employment at recruitment and a follow-up for participants with available data on nature of employment. 14](#_Toc232666949)

[**Supplementary Figure 4.** Participants employment nature at recruitment in the REACT study and at recruitment in the follow-up study by Long COVID status. The plot shows the observed probability of transitioning from one nature of employment to another**.** 15](#_Toc232666950)

[**Supplementary Figure 5.** Estimated odds ratios and 95% confidence interval for the fully-adjusted model relating being in paid work at follow-up and Long COVID status. Reference level for age is the observed median age (52 years). 16](#_Toc232666951)

[**Supplementary Figure 6.** Estimated odds ratios and 95% confidence interval for the fully-adjusted model relating changes in hours of paid work due to ill-health and Long COVID status. Reference level for age is the observed median age (52 years). 17](#_Toc232666952)

[**Supplementary Figure 7.** Estimated odds ratios and 95% confidence interval for the minimally-adjusted model relating being in paid work at follow-up and Long COVID status. Reference level for age is the observed median age (52 years). 18](#_Toc232666953)

[**Supplementary Figure 8.** Estimated odds ratios and 95% confidence interval for the minimally-adjusted model relating changes in hours of paid work due to ill-health and Long COVID status. Reference level for age is the observed median age (52 years). 19](#_Toc232666954)

[**Supplementary Figure 9.** Relative contribution of each variable to model performance in the fully-adjusted model. Panel (A) refers to the model with being in paid work at follow-up as the outcome. Panel (B) refers to the model with changes in hours of work due to physical/mental health as the outcome 20](#_Toc232666955)

[**Supplementary Figure 10.** Symptoms prevalence among participants with Long COVID (resolved or unresolved) and available data on symptoms (N = 2,208). 21](#_Toc232666956)

[**Supplementary Figure 11.** Dendrogram based on hierarchical clustering where different colours represent different clusters. Panel (A) shows the two-cluster solution, panel (B) show the three-cluster solution. Different colours represent different clusters. 22](#_Toc232666957)

[**Supplementary Figure 12.** Stability consensus score for hierarchical clustering and partition around medoids (PAM) clustering by numbers of clusters. 23](#_Toc232666958)

[**Supplementary Figure 13.** Clusters of persistent symptoms lasting at least 12 weeks among participants with resolved and unresolved Long COVID. Clusters were derived using hierarchical clustering. Cluster 1 (N = 1,514) describes a fatigue-predominant Long COVID while Cluster 2 (N = 694) describes a multisystem severe Long COVID. 24](#_Toc232666959)

[**Supplementary Figure 14.** Stability consensus score for hierarchical clustering by number of clusters. Panel (A) refers to the set of participants with persistent symptoms lasting at least 12 weeks, with information on symptoms and COVID-19 history regardless of whether they were in paid work at recruitment in the REACT study. Panel (B) refers to the ser of participants with persistent symptoms lasting at least 12 weeks, who were in paid work and had yet to had COVID-19 at time of recruitment in REACT. 25](#_Toc232666960)

[**Supplementary Figure 15.** Clusters of persistent symptoms lasting at least 12 weeks among participants with resolved and unresolved Long COVID. Participants with information on symptoms were included regardless of whether they were in paid work at time of recruitment in the REACT study (N = 3,075). Clusters were derived using hierarchical clustering. Cluster 1 (N = 1,163) describes a fatigue-predominant Long COVID. Cluster 2 (N = 1,393) describes Long COVID characterised by loss/change of smell and taste. Cluster 3 (N = 519) describes a multisystem severe Long COVID. 26](#_Toc232666961)

[**Supplementary Figure 16.** Clusters of persistent symptoms lasting at least 12 weeks among participants with resolved and unresolved Long COVID. Only participants with information on symptoms, who were in paid employment at baseline and who had yet had to develop their first COVID-19 infection before recruitment in the REACT study are considered (N = 933). Clusters were derived using hierarchical clustering. Cluster 1 (N = 326) describes a fatigue-predominant Long COVID. Cluster 2 (N = 361) describes Long COVID characterised by loss/change of smell and taste. Cluster 3 (N = 246) describes a multisystem severe Long COVID. 27](#_Toc232666962)

[**Supplementary Figure 17.** Clusters of persistent symptoms lasting at least 12 weeks among participants with resolved and unresolved Long COVID. Clusters were derived via the PAM algorithm. Cluster 1 (N = 967) describes a fatigue-predominant Long COVID. Cluster 2 (N = 552) describes Long COVID characterised mainly by loss/change of smell and taste. Cluster 3 (N = 680) describes multisystem severe Long COVID. 28](#_Toc232666963)

[**Supplementary Figure 18.** Cluster memberships overlap between PAM clustering and hierarchical clustering. 29](#_Toc232666964)

**TABLE**

[**Supplementary Table 1.** Socio-demographics characteristics of the REACT sample compared to the ONS estimates for population of England 30](#_Toc232667152)

[**Supplementary Table 2.** Characteristics of the participants included in the analysis by Long COVID status. 31](#_Toc232667153)

[**Supplementary Table 3.** Associations between Long COVID status with being in paid work and changes in hours of work at follow-up according to participants’ gender**.** 32](#_Toc232667154)

[**Supplementary Table 4.** Associations between Long COVID status and clusters of persistent symptoms with being in paid work and changes in hours of work at follow-up according to participants’ socio-economic status measured by IMD quintiles**.** 33](#_Toc232667155)

[**Supplementary Table 5.** Participants characteristics according to cluster membership for the three-cluster solution. Clusters were derived using a hierarchical clustering algorithm. Cluster 1 (N = 1,048) describes a fatigue-predominant Long COVID. Cluster 2 (N = 466) describes Long COVID characterised mainly by loss/change of smell and taste. Cluster 3 (N = 694) describes multisystem severe Long COVID 34](#_Toc232667156)

[**Supplementary Table 6.** Participants characteristics according to cluster membership for the two-cluster solution. Clusters were derived using a hierarchical clustering algorithm. Cluster 1 includes participants with fatigue-predominant Long COVID while cluster 2 includes participants with multisystem severe Long COVID. 36](#_Toc232667157)

[**Supplementary Table 7.** Associations between Long COVID symptoms clusters with being in paid work and changes in hours of work at follow-up in the fully-adjusted model. Symptoms clusters were derived using hierarchical clustering with two cluster solutions 37](#_Toc232667158)

[**Supplementary Table 8.** Frequency and prevalence of Long COVID status among participants with confirmed and suspected infections. 38](#_Toc232667159)

[**Supplementary Table 9.** Associations between Long COVID status with being in paid work and changes in hours of work at follow-up. Long COVID status was computed including both confirmed by test and suspected infections. 38](#_Toc232667160)

[**Supplementary Table 10.** Characteristics of participants included in the sensitivity analyses 39](#_Toc232667161)

[**Supplementary Table 11.**Associations between Long COVID status and symptoms with being in paid work and changes in hours of work at follow-up. All participants, regardless of their baseline employment, in the REACT-LC 2022 follow-up survey with known history of COVID-19 are included (N = 83,648). 41](#_Toc232667162)

[**Supplementary Table 12**.Associations between Long COVID status and symptoms with being in paid work and changes in hours of work at follow-up. Participants are included if they were in paid employment at baseline and had yet to develop their first COVID-19 infection (N = 36,458)**.** 42](#_Toc232667163)

[**Supplementary Table 13.** Associations between Long COVID symptoms clusters with being in paid work and changes in hours of work at follow-up. Symptoms clusters were derived with the PAM algorithm 42](#_Toc232667164)

**SUPPLEMENTARY METHODS**

## **SM1. REACT Sampling Design**

*REACT-1*

The REACT-1 study recruited participants aged 5 and above from those registered with a general practitioner (GP) in England. A new random sample was obtained at each round (for 19 rounds) from data on the NHS register held centrally by NHS Digital.

For the first 11 rounds (May 2020 to May 2021), participants were recruited by:

1. stratifying by lower tier local authority (LTLA), and
2. sampling approximately the same number of participants in each of the 315 LTLA (excluding the Isle of Scilly and combining City of London with Westminster).

For the final seven rounds (May 2021 to March 2022), the sampling procedure was adjusted to select participants randomly in proportion to the LTLA population size. The change was introduced to avoid underrepresentation of urban and inner-city areas, which at the time had the higher infection rate^1^.

*REACT-2*

The REACT-2 study recruited participants aged 18 and above from those registered with a general practitioner (GP) in England. A new random sample was obtained at each round (for 6 rounds) from data on the NHS register held centrally by NHS Digital. As for the first 11 REACT-1 rounds, sampling was stratified by LTLA. Because vaccines were rolled oud in order of decreasing age from December 2020, in the last round (round six, May 2021), sampling was adjusted to achieve a boost of 70,000 people in the age groups 55 to 64 and 65 to 74 years to include additional numbers after their first two vaccination doses^2^.

*REACT 2022 Follow-up Survey*

The 2022 REACT follow-up study aimed to recruit 160,000 adult participants from REACT-1 and REACT-2 studies who agreed to be re-contacted. Based on a 20% response rate, 800,000 REACT-1 and REACT-2 participants were invited based on the following order:

1. Participants in REACT-1 and REACT-2 who previously reported confirmed or suspected COVID-19 infections and symptoms lasting at least 12 weeks (N = 52,501)
2. REACT-1 participants who had tested positive for SARS-CoV-2 (N = 13,482)
3. REACT-2 participants who had tested positive for SARS-CoV- antibodies and had not been vaccinated at the time (N = 85,757)
4. A random sample of all remaining participants not meeting the above criteria (N = 648,260)^3^.

**SM2. Survey Information**

Surveys used in the study can be viewed at the links below:

- REACT-1: [REACT 1 Study Materials | Faculty of Medicine | Imperial College London](https://www.imperial.ac.uk/medicine/research-and-impact/groups/react-study/studies/the-react-1-programme/react-1-study-materials/)
- REACT-2: [The REACT-2 programme | Faculty of Medicine | Imperial College London](https://www.imperial.ac.uk/medicine/research-and-impact/groups/react-study/studies/the-react-2-programme/)
- REACT 2022 follow-up: [REACT Long COVID | Faculty of Medicine | Imperial College London](https://www.imperial.ac.uk/medicine/research-and-impact/groups/react-study/studies/react-long-covid/)

## **SM2. Survey Questions Used to Derive the Outcomes**

Question used to determine whether a participant was still in-paid work at time of the 2022 follow-up was taken directly from the REACT 2022 follow-up and it was phrased as follow:

*EMPL*

At the present are you…? Please select your current job. If more than one applies, please choose the one you do for most hours.

1. Employee in full-time job (30+ hours a week)
2. Employee in part-time job (less than 30 hours a week)
3. Self-employed
4. Government supported training
5. Unemployed and available for work
6. Wholly retired from work
7. Full-time education at school, college or University
8. Looking after home/family
9. Permanently sick/disabled
10. Doing something else
11. Prefer not to say

Participants were labelled as being in paid work if the selected either options 1, 2 or 3.

Question used to determine whether a participant had to change their hours of paid work was taken directly from the REACT 2022 follow-up, and it was phrased as follows:

*LIMITA*

Since March 2020, has your physical or mental health affected the number of hours of paid work that you can do?

1. Yes
2. No
3. Don’t know

This question was asked to every participant regardless of their current employment. None of the participants with a known COVID-19 history answered “Don’t know”, and thus the variable was treated as a binary outcome.

## **SM3. Survey Questions Used to Derive the Long COVID Exposure**

*HADCOVID*

Do you think you have or have ever had the coronavirus (COVID-19)?

1. Yes
2. No
3. Don’t know

*ASK IF HADCOVID = 1*

*HADCOVIDTIMES*

Do you think you have had COVID-19 more than once?

1. Yes
2. Not sure
3. No
4. Prefer not to say

*ASK IF HADCOVID= 1*

*COVIDNUM*

How many times do you think you have had COVID-19? [Insert a number]

*ASK IF HADCOVID = 1 AND HADCOVIDTIMES = 2, 3, 4*

*COVIDA*

Thinking about the first time you had COVID-19, was it…

1. Confirmed by a positive test (swab/PCR/antigen test/lateral flow swab test) (A swab/PCR/antigen test/lateral flow swab test is done by a nasal or throat swab and tests for current COVID-19 infection)
2. Suspected by a doctor but not tested
3. My own suspicions

*ASK IF COVIDA = 1, 2, OR 3*

*COVIDC1*

How severe was your illness when you had COVID-19 the first time?

1. No symptoms
2. Mild symptoms – didn’t affect my daily life
3. Moderate symptoms – some effect on my daily life
4. Severe symptoms – significant effect on my daily life

*ASK IF COVIDC1 = 2, 3 OR 4*

*COVIDSTA1*

When did your first symptoms start when you had COVID-19 for the first time (as best as you can remember)?

DAY/MONTH/YEAR

*ASK IF COVIDC1 = 2, 3, OR 4*

*COVIDEND1*

When did your symptoms finish when you had COVID-19 the first time?

1. DAY/MONTH/YEAR
2. I still have symptoms

*ASK IF HADCOVID = 1 AND HADCOVIDTIMES = 1*

*COVIDA2*

Thinking about the most recent time you had COVID-19, was it…

1. Confirmed by a positive test (swab/PCR/antigen test/lateral flow swab test) (A swab/PCR/antigen test/lateral flow swab test is done by a nasal or throat swab and tests for current COVID-19 infection)
2. Suspected by a doctor but not tested
3. My own suspicions

*ASK IF COVIDA2 = 1, 2, OR 3*

*COVIDC2*

How severe was your illness when you had COVID-19 the most recent time?

1. No symptoms
2. Mild symptoms – didn’t affect my daily life
3. Moderate symptoms – some effect on my daily life
4. Severe symptoms – significant effect on my daily life

*ASK IF COVIDC2 = 2, 3 OR 4*

*COVIDSTA2*

When did your first symptoms start when you had COVID-19 the most recent (as best as you can remember)?

DAY/MONTH/YEAR

*ASK IF COVIDC2 = 2, 3, OR 4*

*COVIDEND2*

When did your symptoms finish when you had COVID-19 the most recent time?

1. DAY/MONTH/YEAR
2. I still have symptoms

## **SM4. Survey Questions Used to Derive Symptoms’ Clusters**

*PERSISTSYMP1*

Please indicate which, if any, persistent symptoms (lasting more than 12 weeks) you think may be linked to you having had COVID-19? Please select all that apply.

1. Fever
2. Persistent cough
3. Shortness of breath (compare to what’s normal for you)
4. Chest pain/tightness
5. Headache
6. Dizziness
7. Mild fatigue (e.g. feeling more tired than normal)
8. Sever fatigue (e.g. inability to get out of bed)
9. Loss of appetite (skipping meals)
10. Joint pain/aches
11. Muscle pain/aches
12. Difficulty thinking or concentrating (“brain fog”)
13. Fast pulse or irregular heartbeat/heart palpitations
14. None of these – FIXED CODE (EXCLUSIVE)

*PERSISTSYMP2*

How about these? Select all that apply.

1. Leg swelling (including due to thrombosis)
2. Sudden swelling of face or lips
3. Red or purples sores/blisters on your feet (including toes)
4. Numbness or tingling somewhere in the body)
5. Skin issues (itchy, scaly, redness, rash)
6. Itchy eyes
7. Loss or change to sense of taste
8. Loss or change to sense of smell
9. Vision issues
10. Ringing in the ears (tinnitus)
11. Hair loss
12. Difficulty sleeping
13. Poor memory
14. Mood swings
15. Anxiety
16. Low mood
17. Other (please write in)
18. None of these
19. Prefer not to say

To identify symptoms’ clusters, we only considered the 29 symptoms listed in the questions. Because “Other” symptoms were varied, we excluded them from the analysis.

## **SM5. Survey Questions Used to Derive Models’ Covariates**

Models’ covariates were derived from information collected in REACT-1 and REACT-2. Nature of employment was derived from:

EMPL

At the present are you…? Please select your current job. If more than one applies, please choose the one you do for most hours.

1. Employee in full-time job (30+ hours a week)
2. Employee in part-time job (less than 30 hours a week)
3. Self-employed
4. Government supported training
5. Unemployed and available for work
6. Wholly retired from work
7. Full-time education at school, college or University
8. Looking after home/family
9. Permanently sick/disabled
10. Doing something else
11. Prefer not to say

In the primary analyses we considered employee in full-time job, part-time job or self-employed only. In the sensitivity analyses that included all participants in the 2022 REACT follow-up with known COVID-19 history (regardless of their employment), we kept the first three options separately and group the remaining eight options (government supported training, unemployed and available to work, wholly retired from work full-time education at school, college or University, looking after home/family, permanently sick/disabled, doing something else, prefer not to say) into a single “Not working/Not specified” category.

For participants who were in a full-time job, a part-time job or were self-employed, type of employment was derived using a combination of the two questions below:

WORKTYPE1

Are you …

1. A healthcare worker with direct patient contact
2. A healthcare worker with no patient contact
3. Working in a care home with direct contact with clients
4. Working in a care home without contact with clients
5. Another essential/key worker (as currently defined by the Government) not in health or social care
6. None of these
7. Don’t know

WORKTYP2

Do you have a job that currently requires you to work outside your home in any of the following public facing roles?

1. Delivering to homes
2. Food retail, other shop work
3. Hospitality – e.g. pubs, restaurants, cafes, hotels
4. Personal care – e.g. hairdresser, beauty therapist, personal trainer
5. Policing, prisons, fire & rescue, coastguard
6. Public transport (including taxis)
7. Education, school, nursery
8. Childcare
9. Armed forces
10. Another public facing role (please specify)
11. I work outside my home but not in a public facing role
12. No, not currently required to work outside my home at all

Based on their answers participants were grouped as follows:

1. Health care: WORKTYP1 = “A health care worker with direct patient contact” OR WORKTYP1 = “A health care worker without direct patient contact”
2. Care home: WORKTYP1 = “A care home worker with direct contact with clients” OR WORKTYP1 = “A care home worker without direct contact to clients”
3. Business and service: WORKTYP2 = “Food retail, other shop work”, OR WORKTYP2 = “Personal care – e.g. hairdresser, beauty therapist, personal trainer” OR WORKTYP2 = “Hospitality – e.g., pubs, restaurants, café, hotels”
4. Child-related: WORKTYP2 = “Education, school, nursery” OR WORKTYP2 = “Childcare”
5. Logistic and security: WORKTYP2 = “Delivering to homes” OR WORKTYP2 = “Policing, prisons, fire & rescue, coastguard” OR WORKTYP2 = “Public transport (including taxis)” OR WORKTYP2 = “Armed forces”
6. Other essential worker: WORKTYP1 = “Another essential/key worker (as currently defined by the Government) not in health or social care” AND WORKTYP1 = “Another public facing role (please specify)”
7. Work at home/not in a public facing role: WORKTYP2 = “I work outside my home but not in a public facing role” OR WORKTYP2 = “No, not currently required to work outside my home at all”.

The other relevant questions are listed below:

*DOB*

What is your date of birth?

1. DAY/MONTH/YEAR
2. Prefer not to say

*SHIELD2*

Are you taking specific precautions because you are concerned that you will become severely ill with COVID-19?

1. Yes
2. No

*SICKPAY3*

If you were off work for two weeks due to illness, how serious would the financial impact be on your household?

1. Very serious
2. Fairly serious
3. Not very serious
4. Not at all serious
5. Prefer not to say

*EDUC*

What is your highest educational qualification? This means educational, professional, vocational, or other work-related qualifications for which you received a certificate. Please select one answer

1. Degree level or above
2. Other Higher Education below degree level
3. A levels, NVQ level 3 and equivalent (includes AS level, SVQ and GNVQ level 3, BTEC National)
4. GCSE/O level grade A*-C or 4-9, NDQ level 2 and equivalents (incudes SVQ and GNVQ level 2, BTEC first or general diploma
5. Qualifications at level 1 and below (includes GCSE or O level below grade C or 4, CSE below grade 1, NVQ, SVQ, GNVQ level 1, BTEC first or general certificate)
6. Another type of qualification (includes other vocational or professional or foreign qualifications)
7. No qualifications

*HEALTHA*

Do you currently have any of the following? Please tick all that apply

1. Organ transplant recipient
2. Diabetes (type I, type II or gestational)
3. Heart disease or heart problems
4. Hypertension (high blood pressure)
5. Stroke
6. Kidney disease
7. Liver disease
8. Anaemia
9. Asthma
10. Other lung condition (such as COPD, bronchitis or emphysema)
11. Cancer
12. Condition affecting the brain and nerves (e.g. Dementia, Parkinson’s, Multiple Sclerosis)
13. A weakened immune system/reduced ability to deal with infections (as a result of a disease or treatment)
14. Depression
15. Anxiety
16. Psychiatric disorder
17. None of these

*ETHINC*

Which of the following best describes your ethnic group or background?

*White*

1. English / Welsh / Scottish / Northern Irish / British
2. Irish
3. Gypsy or Irish Traveller
4. Any other White background

*Mixed*

1. White and Black Caribbean
2. White and Black African
3. White and Asian
4. Any other Mixed/Multiple ethnic background

*Asian*

1. Indian
2. Pakistani
3. Bangladeshi
4. Chinese
5. Any other Asian background

*Black*

1. African
2. Caribbean
3. Any other Black / African / Caribbean background

*Other*

1. Arab
2. Any other ethnic group
3. Prefer not to say

## **SM6. Multiple Imputation**

Multiple imputation was performed using the mice package in R.^4^ Missing data were imputed using predictive mean matching with five nearest donors. In total, 10 imputed datasets were created. For each imputation, the chained-equations sampler was run for 50 iterations. The imputation model included all covariates in the final analytic model, the outcome variables, and any auxiliary variables that could help with predicting missing information. All imputations were generated under a fixed random seed to guarantee reproducibility.

*Analysis with Long COVID status as the exposure*

When the exposure of interest was Long COVID status, the imputation model included:

- Outcomes:
  - “In paid work” at follow-up (yes/no)
  - change hours of paid work (yes/no)
- Model covariates:
  - type of employment at recruitment in REACT (business and service; care home; child-related; health care workers; logistic and security; other essential worker; work at home/not public facing)
  - nature of employment at recruitment in REACT (full-time; part-time; self-employed)
  - gender (male/female)
  - age (years)
  - Long COVID status (no COVID-19; asymptomatic or resolved short COVID-19 less than 4 weeks; resolved short COVID-19 >4 to <12 weeks; resolved Long COVID; unresolved Long COVID)
  - number of comorbidities (0, 1, 2+)
  - index of multiple deprivation (IMD) quintile
  - taking precaution due to concern of becoming ill with COVID-19 (yes/no)
  - months of follow-up
  - ethnicity (Asian; Black; Mixed; White; Other)
  - financial impact of two weeks off work due to illness (not serious at all; note very serious; fairly serious; very serious)
  - education level (degree level or higher; other higher qualifications below degree level; A-levels, NVQ levels 3 and equivalents; GCSE/O-levels; qualification at level 1 and below; other qualification; no qualification).
- Auxiliary variables:
  - COVID-19 symptoms (no symptoms, mild symptoms, moderate symptoms, severe symptoms)
  - changes in kind of paid work (yes/no)

*Sensitivity Analyses and Analysis with symptoms cluster as the exposure*

Because the sensitivity analyses and the analysis with symptoms cluster as the exposure involved a different analytic sample size and a potentially modified missing data pattern, to avoid any congeniality issues between the imputation model and the analysis model, rather than re-using the imputations generated under the primary sample, we re-estimated the entire imputation model for the sensitivity analyses and the analysis with symptoms cluster as the outcome. The imputed datasets were generated using the same specifications (10 imputation, predictive mean matching with five donors, and 50 iterations) and set of variables. When the exposure of interest was symptoms cluster, the imputation model included the same variables model listed above together with an additional variable indicating symptoms cluster (C1; C2; C3).

## **SM7. Cluster Stability**

Clusters stability was assessed with resampling-based consensus clustering. Specifically, we 1) took 1,000 random subsamples of the data, 2) applied the same hierarchical clustering algorithm to each subsample, 3) computed the probability that items clustered together across subsamples, 4) summarised these probabilities in a consensus score measuring how far they deviated from random clustering. Higher scores indicate stronger cluster stability.^5^

## **SM8. Partition Around Medoid (PAM) Algorithm**

PAM identifies the most representative data point in each cluster, assigns participants to clusters based on their distance from these representative points, and iteratively refines the selection to minimize within-cluster dissimilarity. We defined dissimilarity based on the Euclidean distance, evaluated two to ten clusters, and determined the optimal number using consensus score and the average silhouette width**.**^6^

**FIGURES**

**Supplementary Figure 1**. **Pipeline for the computation of the Long COVID status exposure**


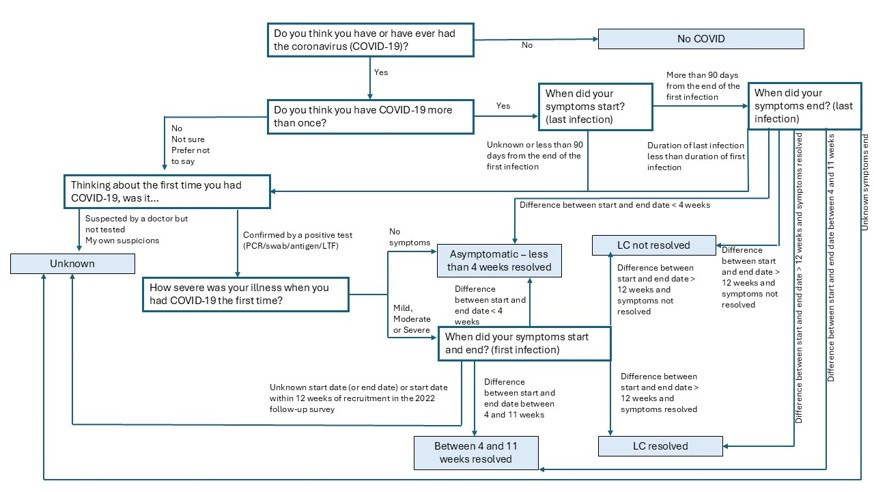
**Note:** When participants had two or more infections, we took the longest COVID-19 infection to derive their Long COVID status. If a participant did not have any symptom during their first and most recent infection, they were classified as asymptomatic – less than 4 weeks resolved

**Supplementary Figure 2. Directed Acyclic Graph (DAG) showing the variables considered when modelling the relationship between Long COVID status and employment outcomes**


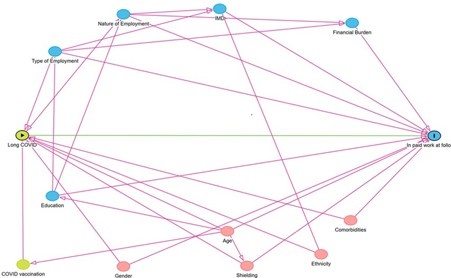


**Note**: we use the same DAG for both outcomes studied (being in paid work at time of follow-up and changes in hours of paid work). The minimally adjusted set of confounders identified by the software was: age, number of comorbidities, education level, ethnicity, gender, type of employment, nature of employment and shielding, Shielding indicates any precautions taken at time of recruitment due to COVID-19 concerns.

**Supplementary Figure 3. Comparison of employment at recruitment and a follow-up for participants with available data on nature of employment.**


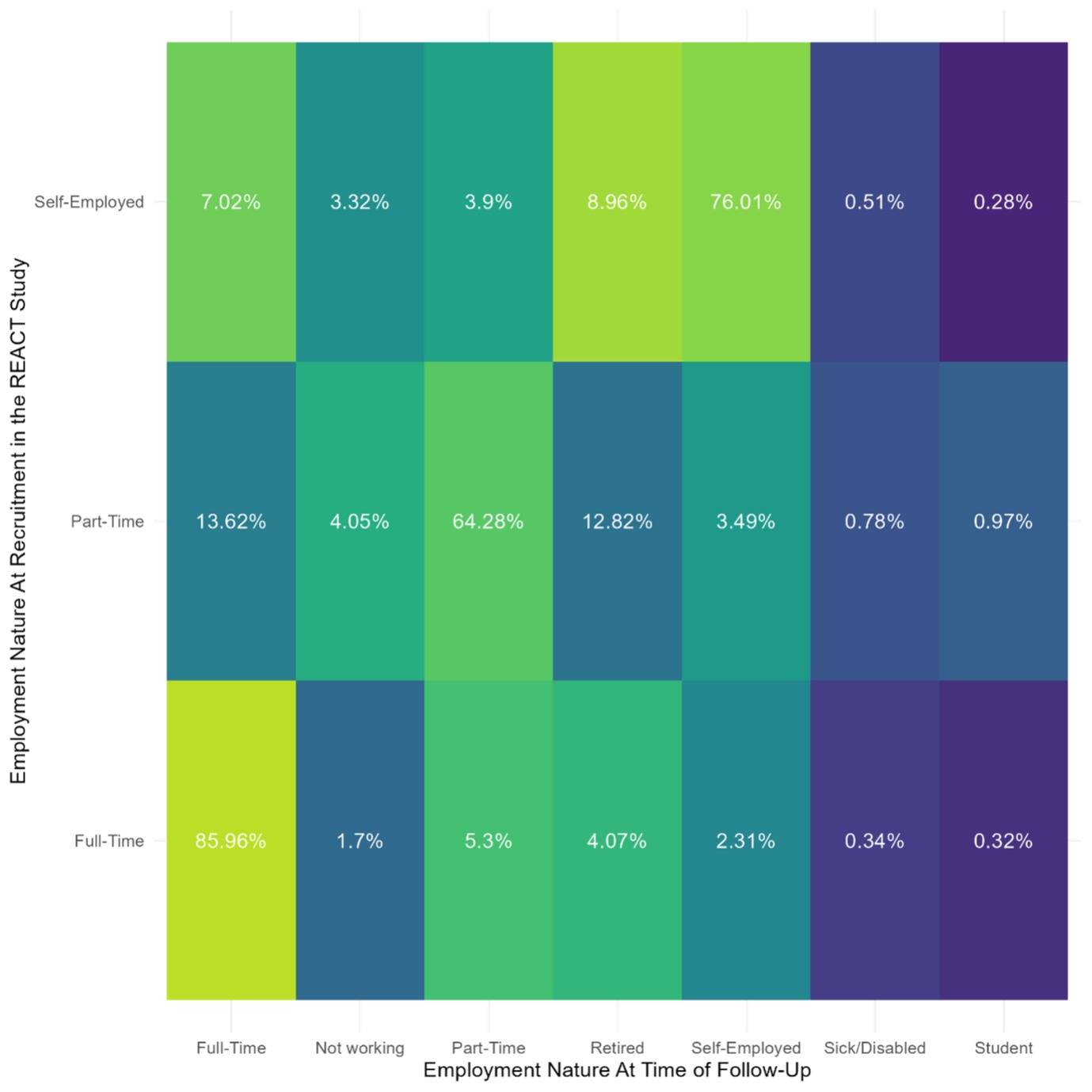


**Supplementary Figure 4. Participants employment nature at recruitment in the REACT study and at recruitment in the follow-up study by Long COVID status. The plot shows the observed probability of transitioning from one nature of employment to another.**


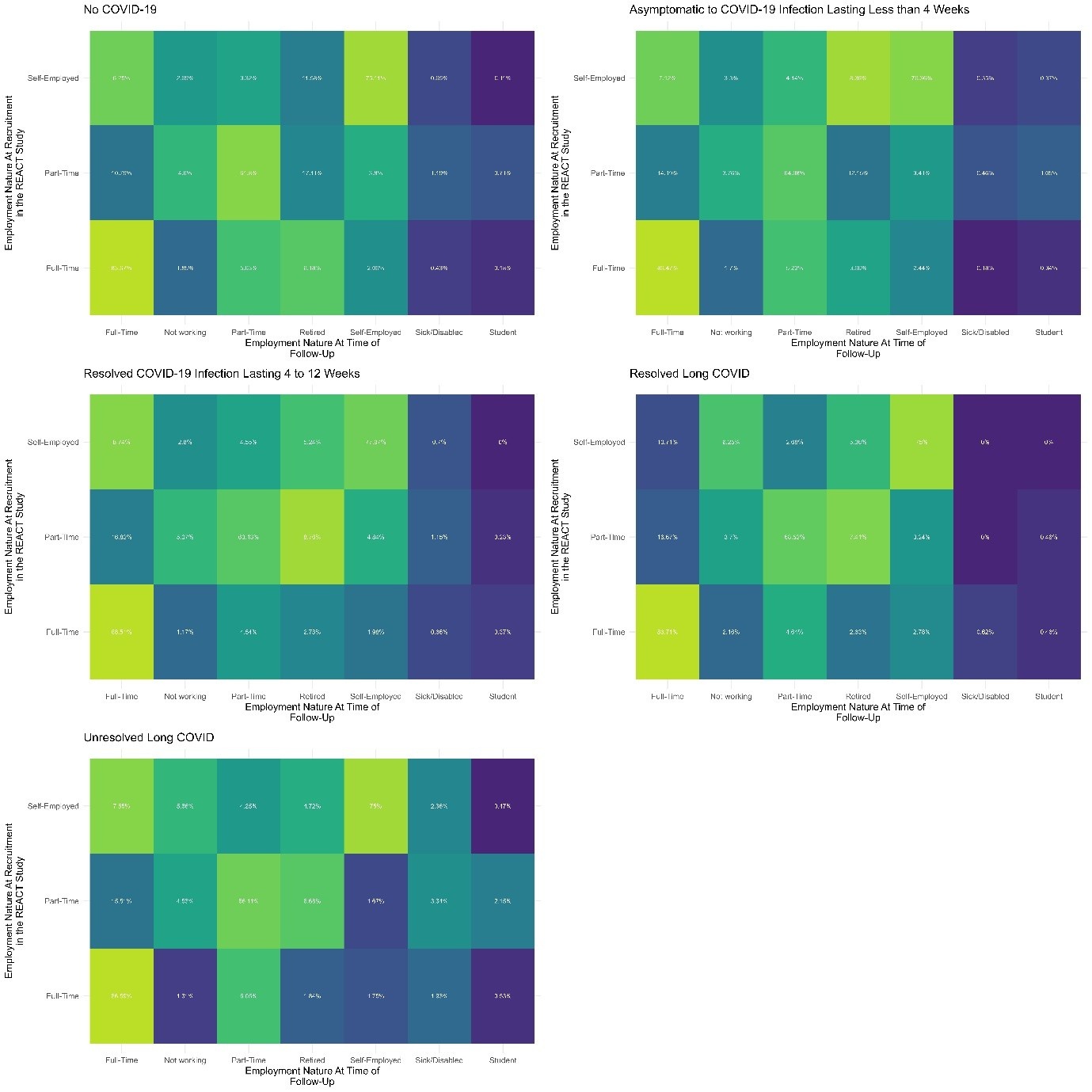


**Supplementary Figure 5. Estimated odds ratios and 95% confidence interval for the fully-adjusted model relating being in paid work at follow-up and Long COVID status. Reference level for age is the observed median age (52 years).**

*
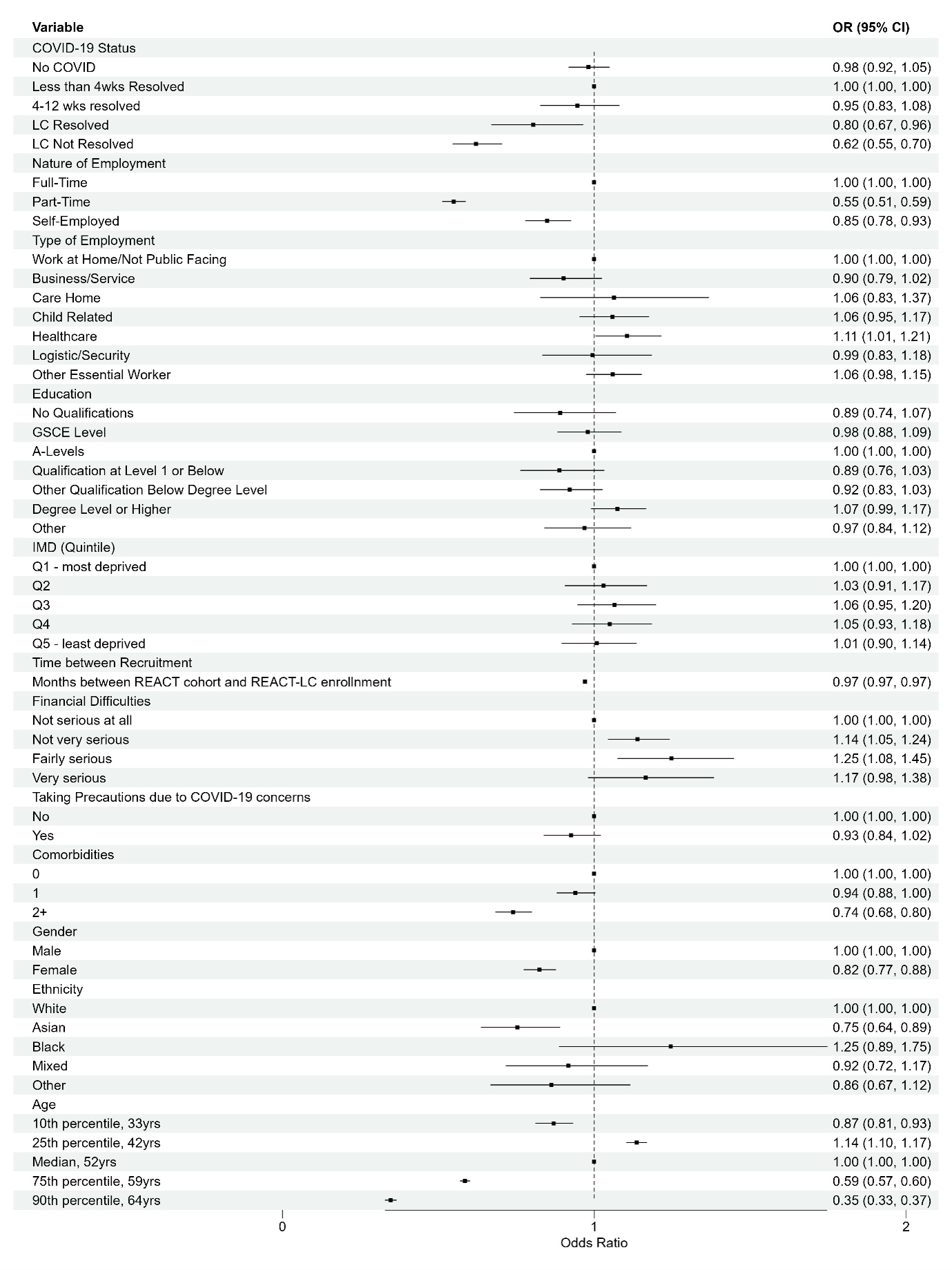
*

**Supplementary Figure 6. Estimated odds ratios and 95% confidence interval for the fully-adjusted model relating changes in hours of paid work due to ill-health and Long COVID status. Reference level for age is the observed median age (52 years).**

**
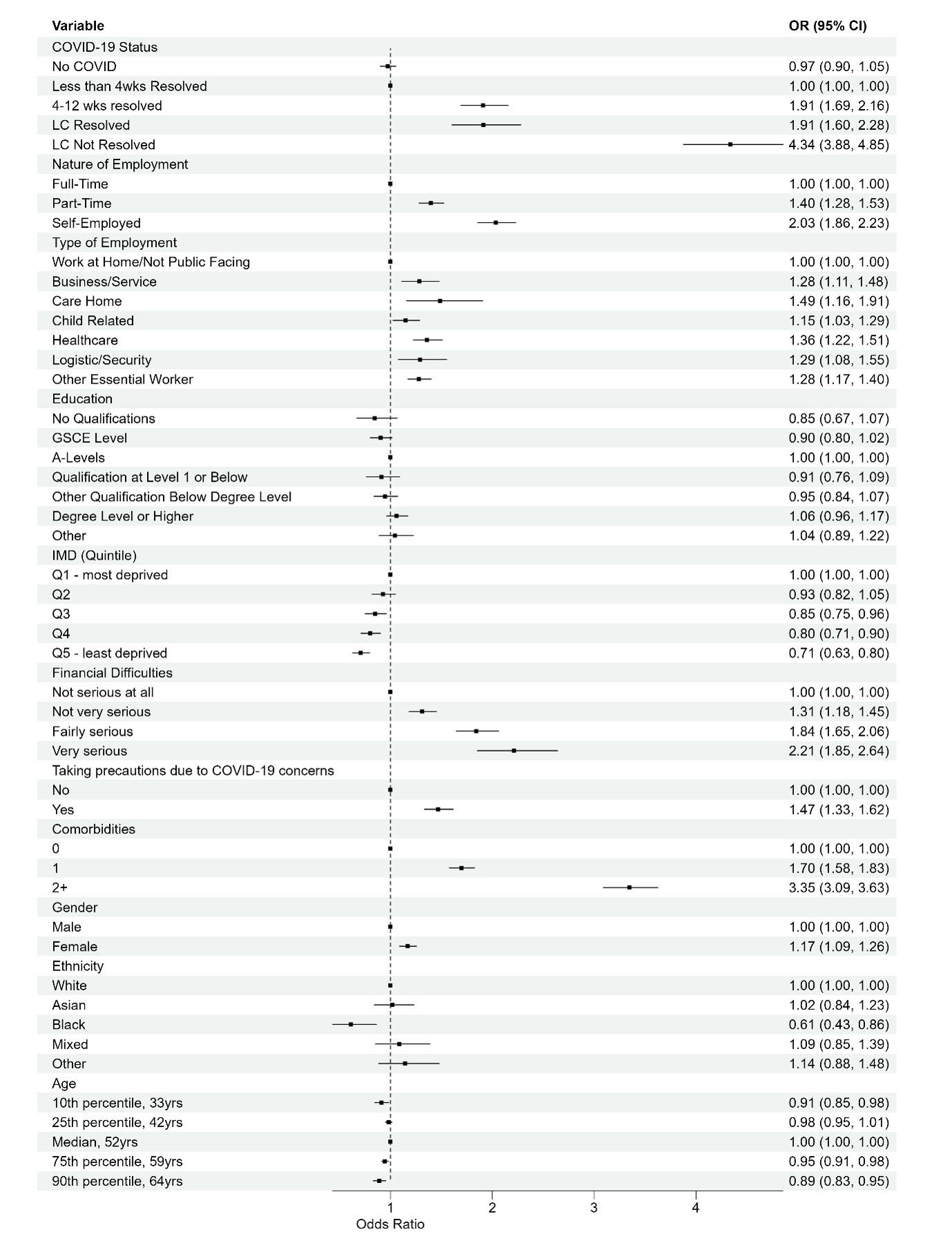
**

**Supplementary Figure 7. Estimated odds ratios and 95% confidence interval for the minimally-adjusted model relating being in paid work at follow-up and Long COVID status. Reference level for age is the observed median age (52 years).**


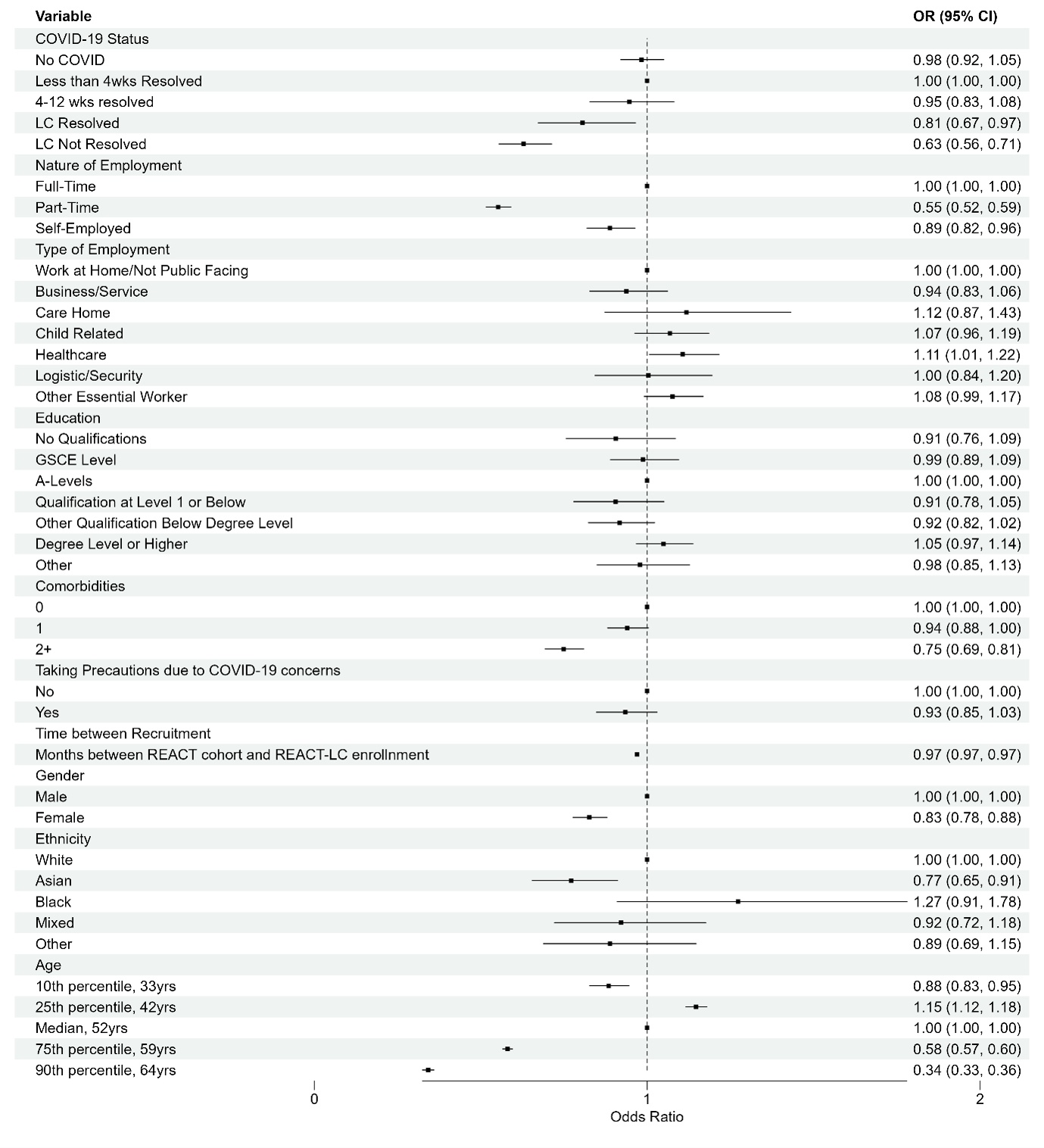


**Supplementary Figure 8. Estimated odds ratios and 95% confidence interval for the minimally-adjusted model relating changes in hours of paid work due to ill-health and Long COVID status. Reference level for age is the observed median age (52 years).**


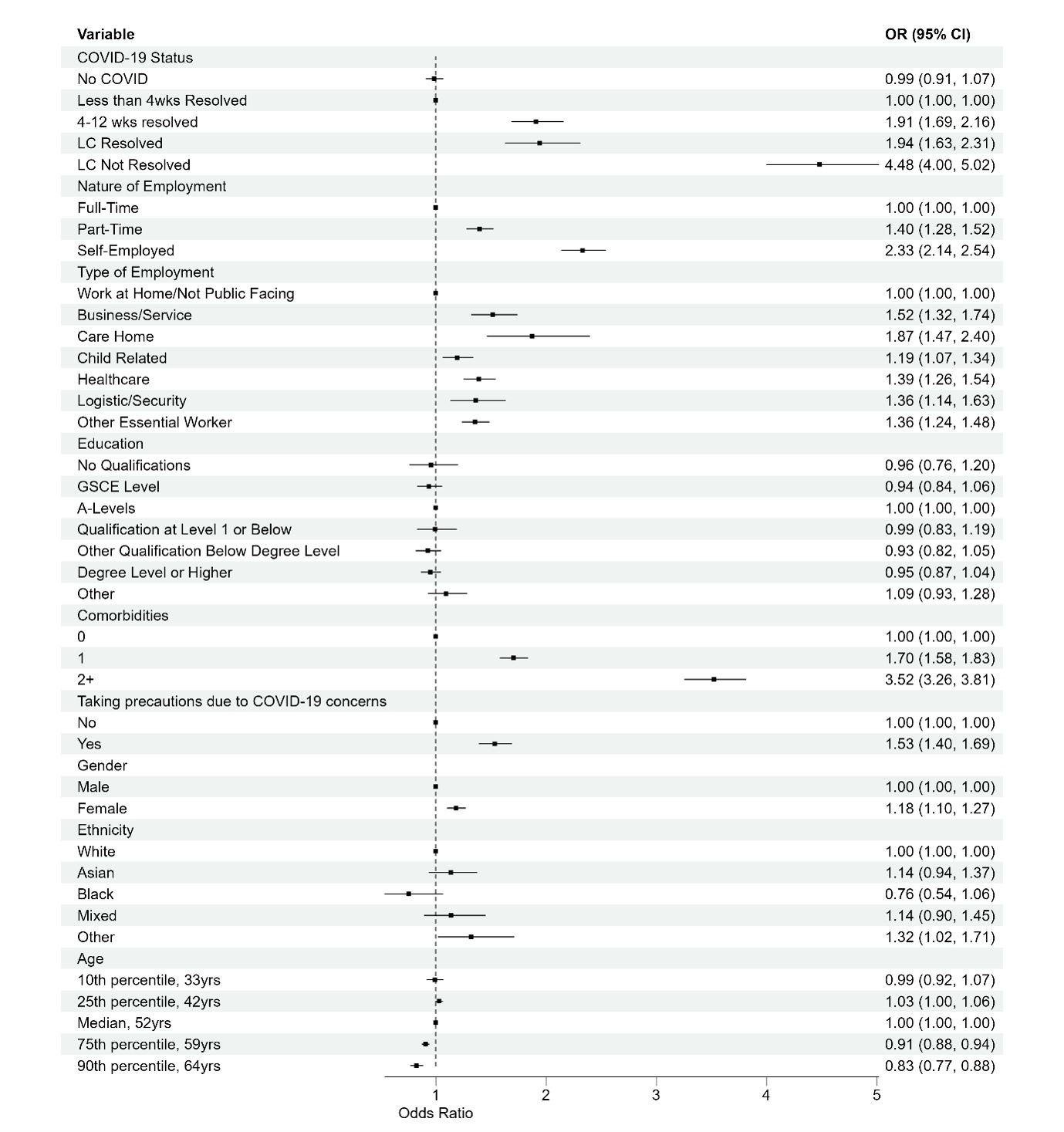


**Supplementary Figure 9. Relative contribution of each variable to model performance in the fully-adjusted model. Panel (A) refers to the model with being in paid work at follow-up as the outcome. Panel (B) refers to the model with changes in hours of work due to physical/mental health as the outcome**


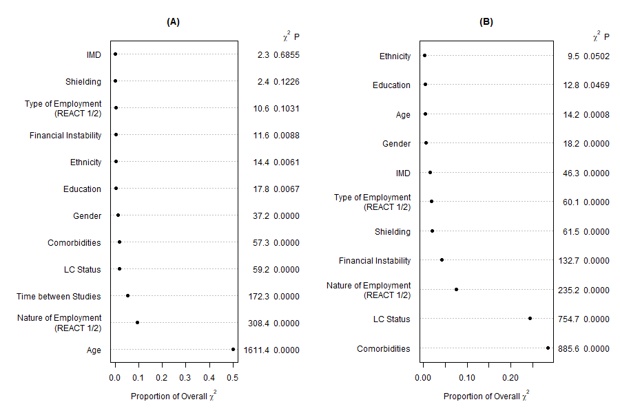


**Supplementary Figure 10. Symptoms prevalence among participants with Long COVID (resolved or unresolved) and available data on symptoms (N = 2,208).**


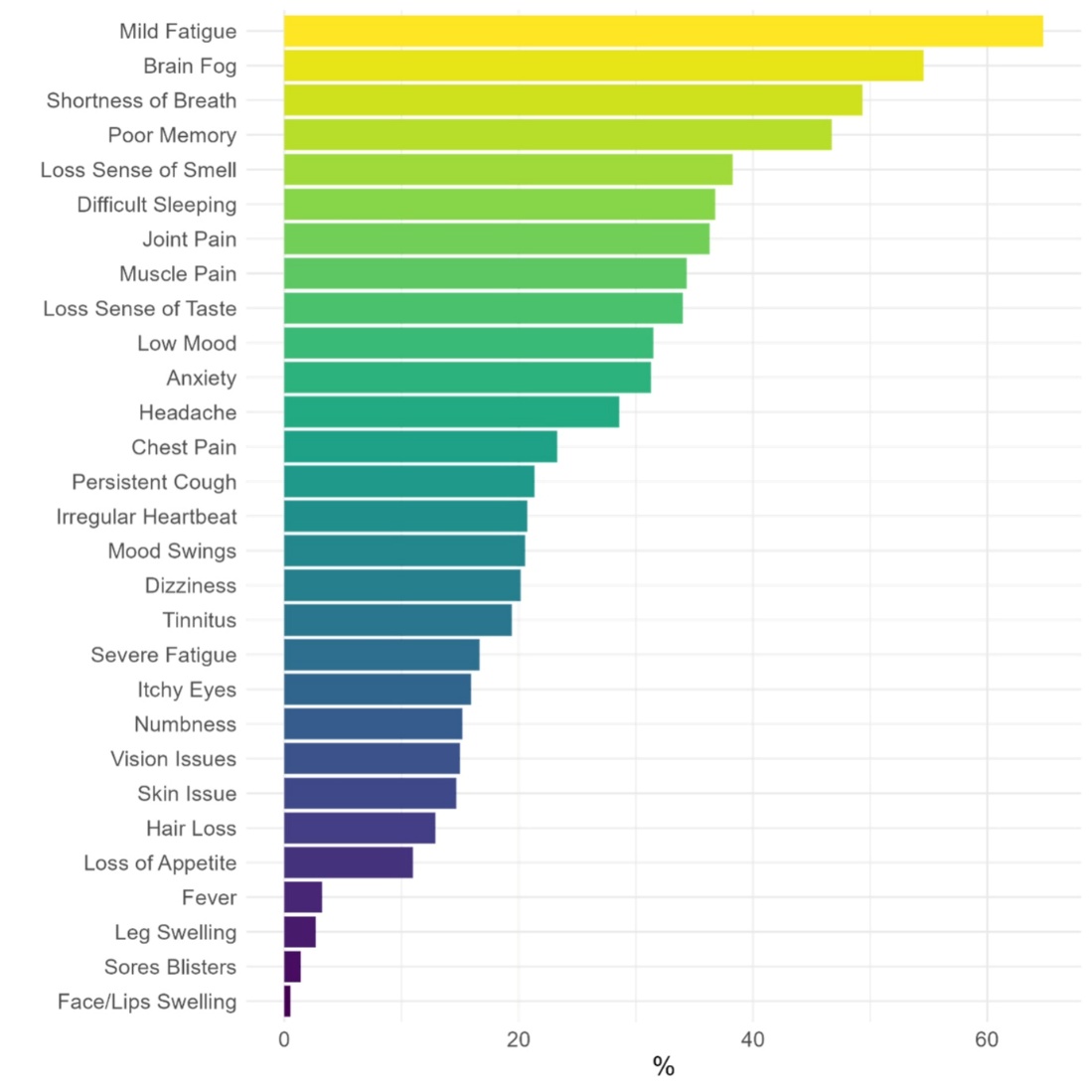


**Supplementary Figure 11. Dendrogram based on hierarchical clustering where different colours represent different clusters. Panel (A) shows the two-cluster solution, panel (B) show the three-cluster solution. Different colours represent different clusters.**


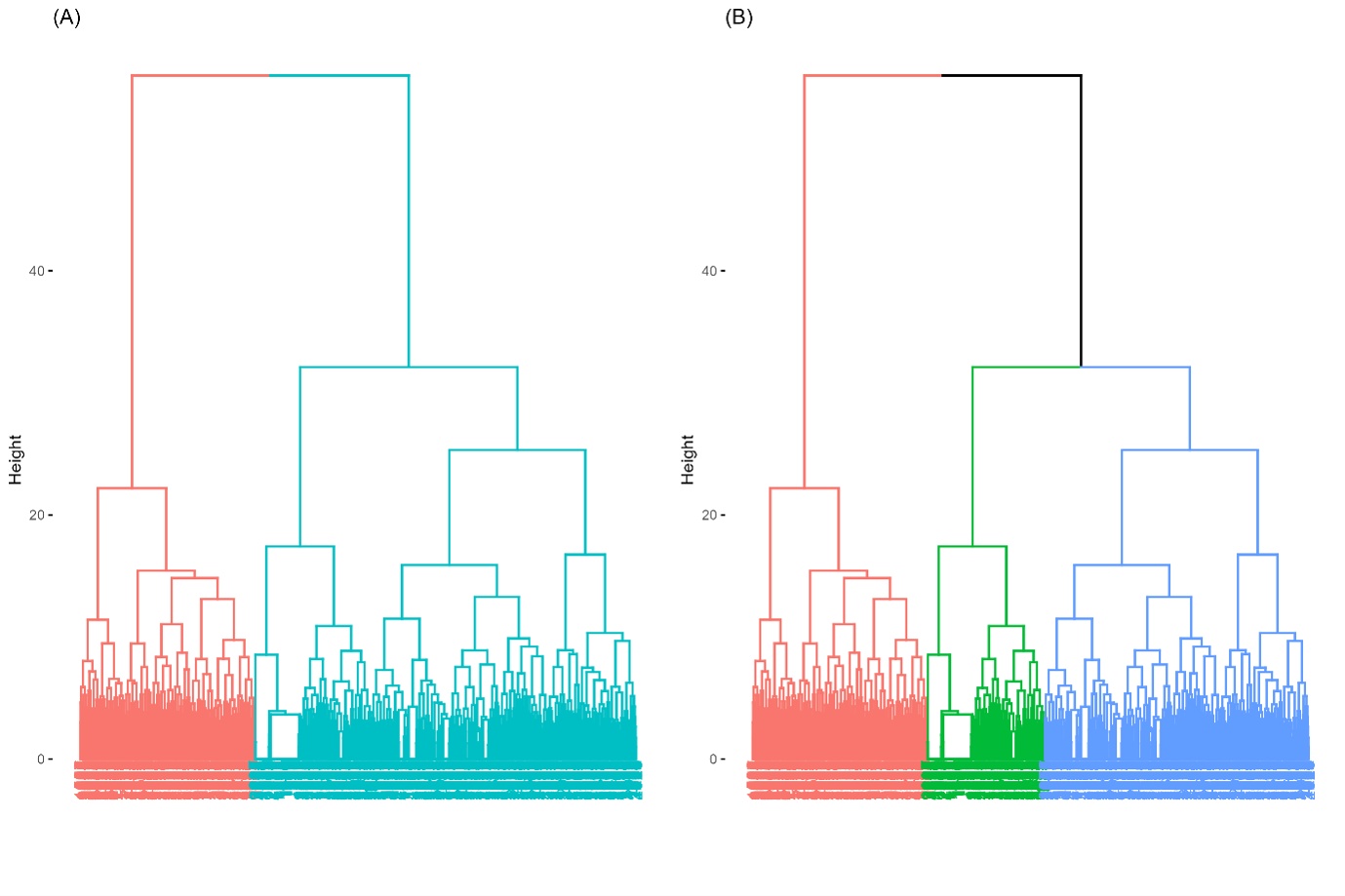


**Supplementary Figure 12. Stability consensus score for hierarchical clustering and partition around medoids (PAM) clustering by numbers of clusters.**

**
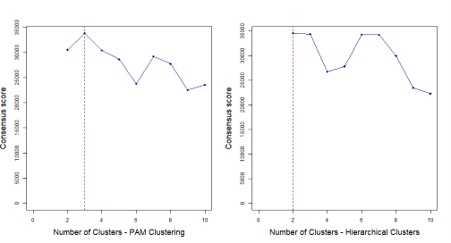
**

**Supplementary Figure 13. Clusters of persistent symptoms lasting at least 12 weeks among participants with resolved and unresolved Long COVID. Clusters were derived using hierarchical clustering. Cluster 1 (N = 1,514) describes a fatigue-predominant Long COVID while Cluster 2 (N = 694) describes a multisystem severe Long COVID.**


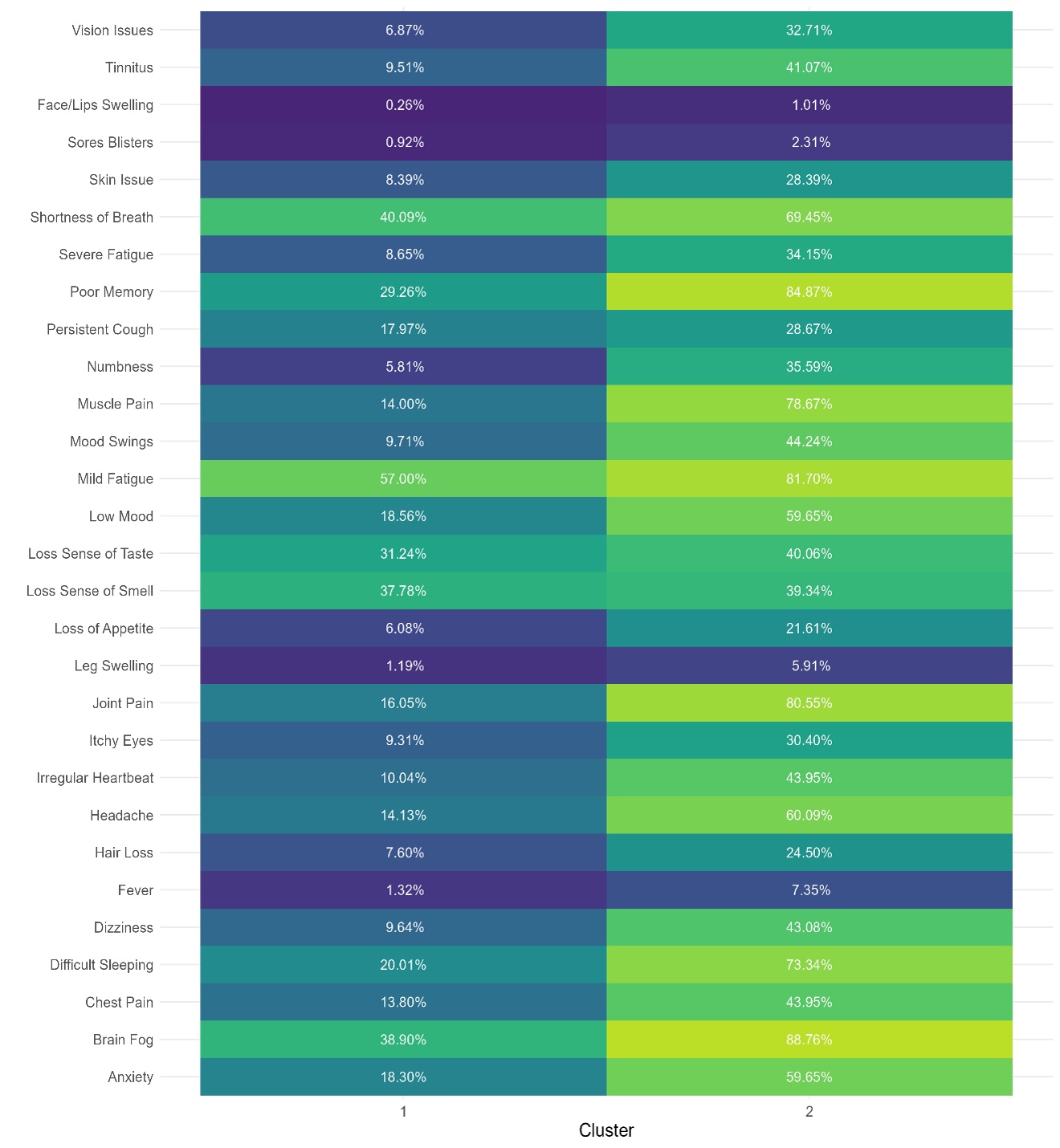


**Supplementary Figure 14. Stability consensus score for hierarchical clustering by number of clusters. Panel (A) refers to the set of participants with persistent symptoms lasting at least 12 weeks, with information on symptoms and COVID-19 history regardless of whether they were in paid work at recruitment in the REACT study. Panel (B) refers to the ser of participants with persistent symptoms lasting at least 12 weeks, who were in paid work and had yet to had COVID-19 at time of recruitment in REACT.**


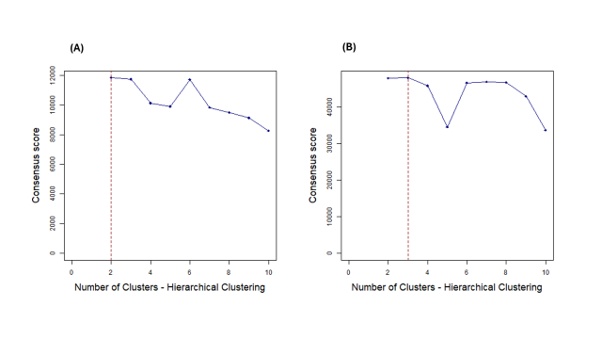


**Supplementary Figure 15. Clusters of persistent symptoms lasting at least 12 weeks among participants with resolved and unresolved Long COVID. Participants with information on symptoms were included regardless of whether they were in paid work at time of recruitment in the REACT study (N = 3,075). Clusters were derived using hierarchical clustering. Cluster 1 (N = 1,163) describes a fatigue-predominant Long COVID. Cluster 2 (N = 1,393) describes Long COVID characterised by loss/change of smell and taste. Cluster 3 (N = 519) describes a multisystem severe Long COVID.**


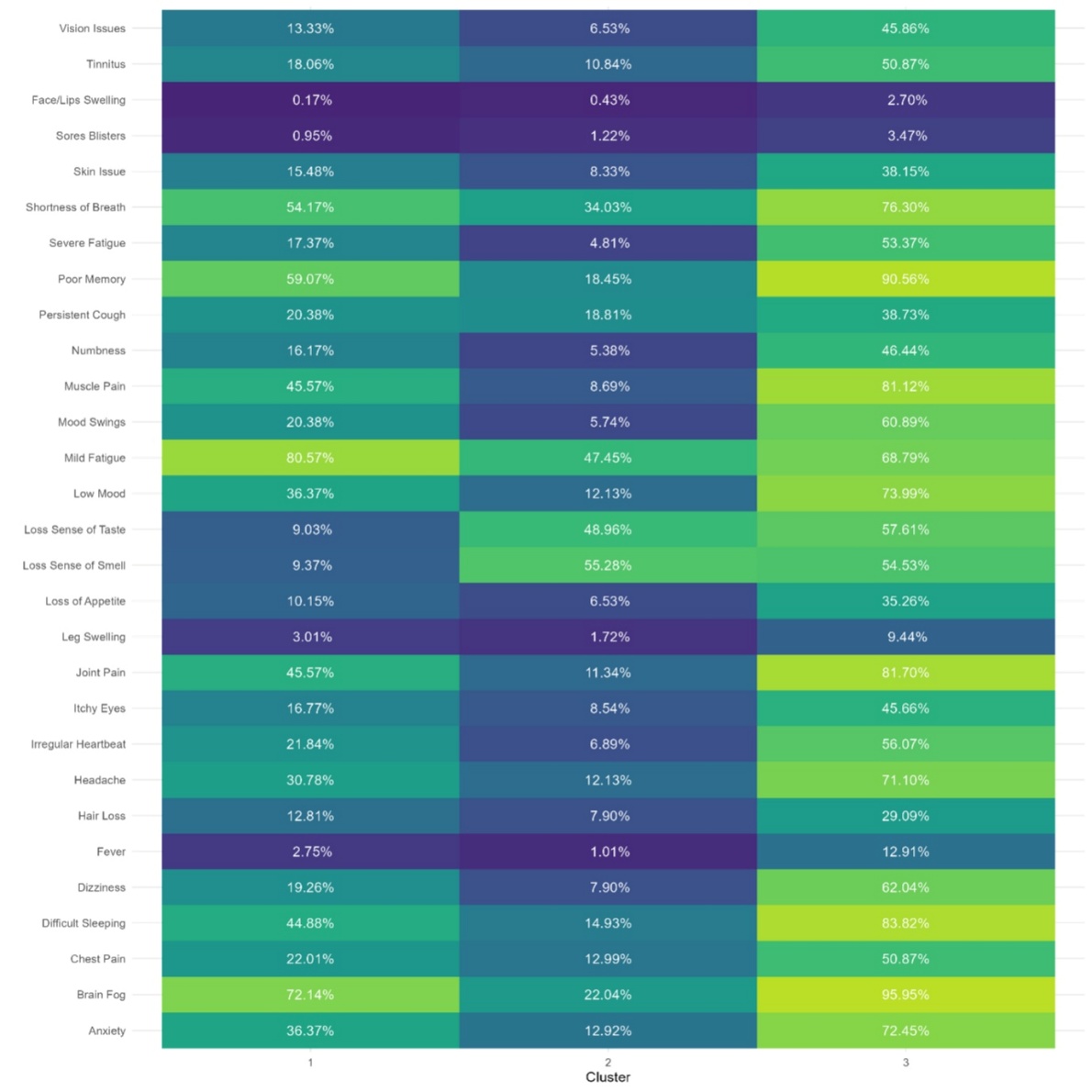


**Supplementary Figure 16. Clusters of persistent symptoms lasting at least 12 weeks among participants with resolved and unresolved Long COVID. Only participants with information on symptoms, who were in paid employment at baseline and who had yet had to develop their first COVID-19 infection before recruitment in the REACT study are considered (N = 933). Clusters were derived using hierarchical clustering. Cluster 1 (N = 326) describes a fatigue-predominant Long COVID. Cluster 2 (N = 361) describes Long COVID characterised by loss/change of smell and taste. Cluster 3 (N = 246) describes a multisystem severe Long COVID.**


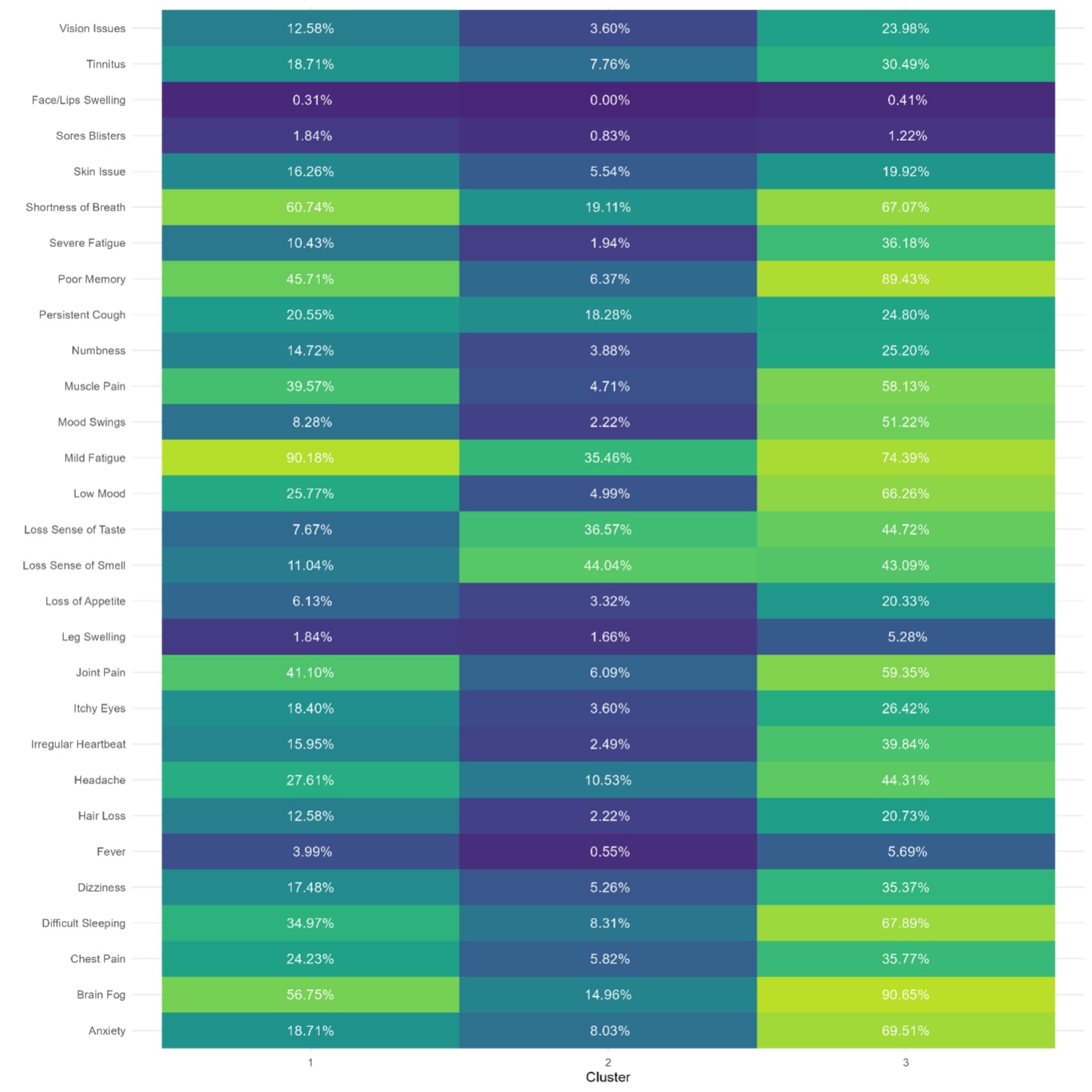


**Supplementary Figure 17. Clusters of persistent symptoms lasting at least 12 weeks among participants with resolved and unresolved Long COVID. Clusters were derived via the PAM algorithm. Cluster 1 (N = 967) describes a fatigue-predominant Long COVID. Cluster 2 (N = 552) describes Long COVID characterised mainly by loss/change of smell and taste. Cluster 3 (N = 680) describes multisystem severe Long COVID.**


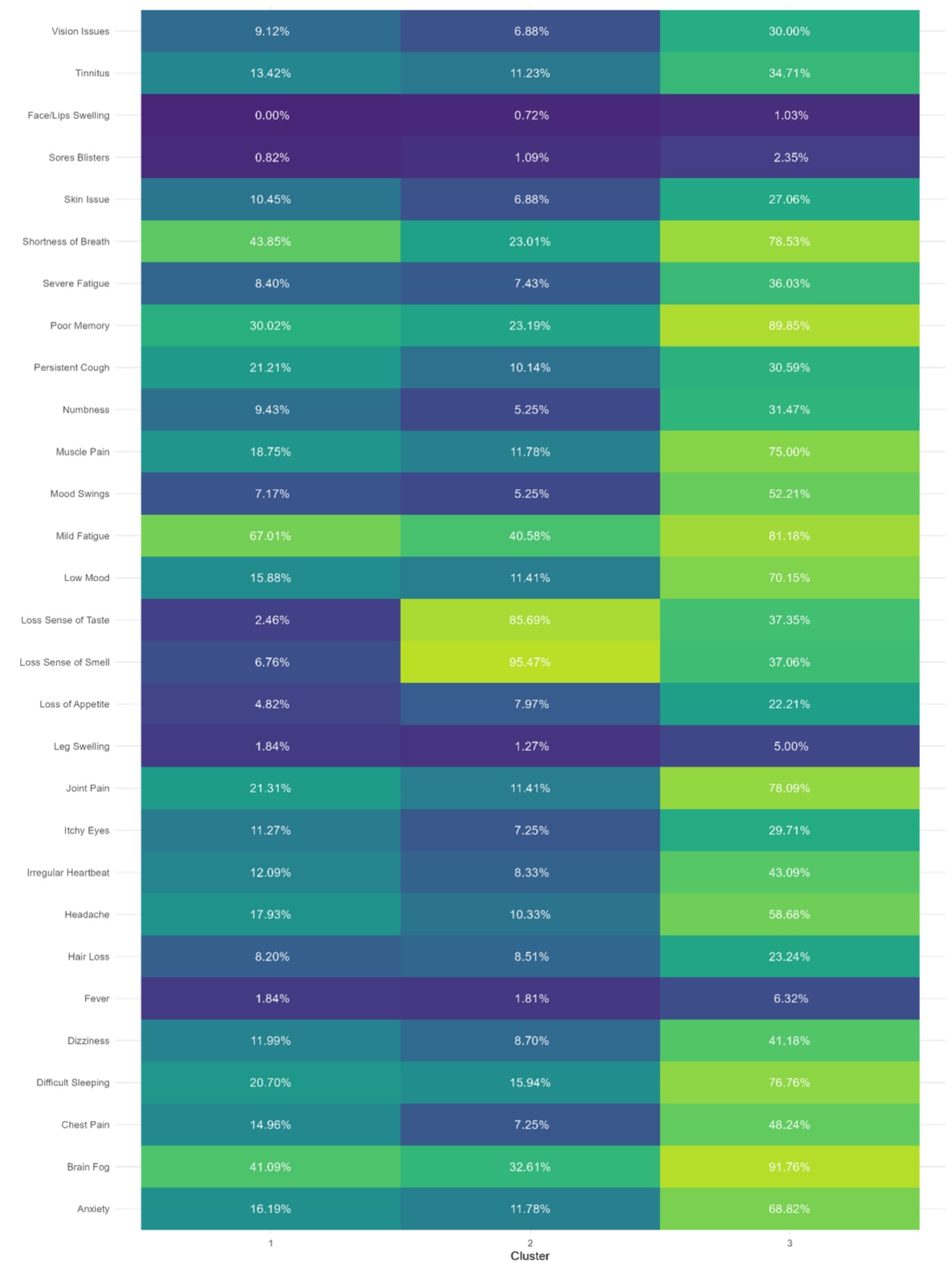


**Supplementary Figure 18. Cluster memberships overlap between PAM clustering and hierarchical clustering.**


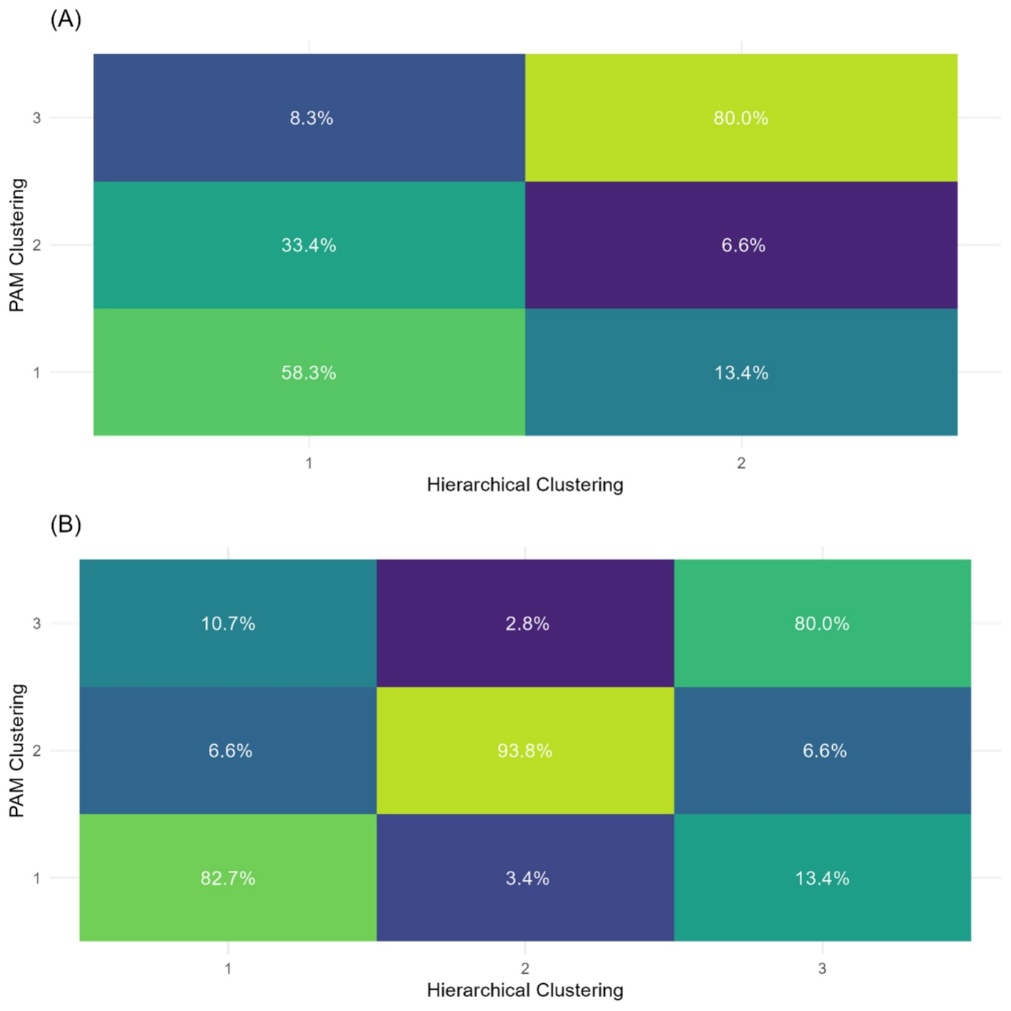


**TABLES**

**Supplementary Table 1. Socio-demographics characteristics of the REACT sample compared to the ONS estimates for population of England**

|  | **England - 2021 (18+ population)**^7–9^ ***** | **REACT-1 and REACT-2 (18+ population) **** | **REACT-LC Waves 3 and 4 Invited** | **REACT-LC Waves 3 and 4 Responders** | **REACT-LC Waves 3 and 4 Responders Included in the Final Sample ***** |
| --- | --- | --- | --- | --- | --- |
| **N** | 43,820,356 | 3,009,386 | 300,656 | 107,029 | 45,864 |
| **Sex** |  |  |  |  |  |
| Male | 48·3% | 44·9% | 43·2% | 41·3% | 40·0% |
| Female | 51·7% | 55·1% | 56·8% | 58·7% | 60·0% |
| **Age (years)** |  |  |  |  |  |
| 18 – 24 | 10·5% | 5·7% | 5·0% | 2·8% | 1·9% |
| 25 – 34 | 16·8% | 12·2% | 10·7% | 5·0% | 10·5% |
| 35 – 44 | 16·3% | 15·3% | 15·1% | 11·5% | 17·7% |
| 45 – 54 | 16·7% | 18·4% | 18·8% | 17·2% | 21·3% |
| 55 – 64 | 16·0% | 21·4% | 22·4% | 25·8% | 32·1% |
| 65 – 74 | 12·6% | 18·4% | 18·7% | 24·8% | 9·3% |
| 75+ | 11·1% | 8·5% | 9·3% | 10·9% | 1·1% |
| **Ethnicity** |  |  |  |  |  |
| Asian | 8·9% | 4·5% | 3·4% | 2·7% | 3·0% |
| Black | 3·8% | 1·4% | 0·9% | 0·8% | 0·9% |
| Mixed | 1·9% | 1·3% | 1·3% | 1·2% | 1·4% |
| Other | 2·1% | 0·9% | 1·2% | 1·2% | 1·2% |
| White | 83·3% | 91·8% | 93·1% | 94·2% | 94·0% |
| **Region** |  |  |  |  |  |
| Northeast | 4·8% | 3·8% | 3·9% | 3·9% | 3·6% |
| Northwest | 13·2% | 11·9% | 11·6% | 10·9% | 10·7% |
| Yorkshire and the Humber | 9·83% | 6·7% | 7·4% | 7·4% | 7·0% |
| East Midlands | 8·71% | 12·7% | 11·6% | 11·1% | 10·9% |
| West Midlands | 10·4% | 9·4% | 9·4% | 9·1% | 8·7% |
| East of England | 11·1% | 14·4% | 13·7% | 13·8% | 14·0% |
| London | 15·4% | 9·9% | 11·0% | 10·9% | 12·6% |
| Southeast | 16·3% | 21·7% | 21·3% | 21·9% | 22·1% |
| Southwest | 10·2% | 9·6% | 10·3% | 10·9% | 10·4% |
| **IMD Quintile** |  |  |  |  |  |
| Q1 – most deprived | 19·1% | 11·2% | 9·8% | 8·4% | 9·0% |
| Q2 | 20·3% | 16·8% | 15·9% | 15·1% | 16·0% |
| Q3 | 20·5% | 21·5% | 21·5% | 21·5% | 22·0% |
| Q4 | 20·2% | 24·2% | 24·8% | 25·3% | 25·0% |
| Q5 – least deprived | 19·8% | 26·4% | 28·0% | 29·7% | 28·0% |
| **Long COVID **** | 3·1% | 1·7% | 6·6% | 5·1% | 6·6% |

****** REACT 1 and 2 participants included in the sample used to determine those invited in the REACT 2022 follow-up survey

****** REACT-LC participants from waves 3 and 4 who were employed (full-time, part-time, self-employed) at time of recruitment in REACT and had a known history of COVID-19. ** Long COVID defined as persistent symptoms lasting longer than 12 weeks

ONS estimates for England were derived from: <https://www.ons.gov.uk/peoplepopulationandcommunity/populationandmigration/populationestimates/datasets/lowersuperoutputareamidyearpopulationestimatesnationalstatistics> (population estimates mid-2021 to mid-2022), [Ethnic group by age and sex in England and Wales - Office for National Statistics](https://www.ons.gov.uk/peoplepopulationandcommunity/culturalidentity/ethnicity/datasets/ethnicgroupbyageandsexinenglandandwales) (ethncity data), and [Prevalence of ongoing symptoms following coronavirus (COVID-19) infection in the UK - Office for National Statistics](https://www.ons.gov.uk/peoplepopulationandcommunity/healthandsocialcare/conditionsanddiseases/bulletins/prevalenceofongoingsymptomsfollowingcoronaviruscovid19infectionintheuk/2february2023) (Long COVID). All accessed via web on April 20, 2026.

**Supplementary Table 2. Characteristics of the participants included in the analysis by Long COVID status.**

|  | No COVID  (N = 10,619) | Asymptomatic or resolved short COVID-19 <4 weeks  (N = 30,008) | Resolved COVID (4 – 11 weeks)  (N = 2,217) | Resolved Long COVID  (N = 1,053) | Unresolved Long COVID  (N = 1,967) |
| --- | --- | --- | --- | --- | --- |
| **Employment Nature at Recruitment in REACT** |  |  |  |  |  |
| Full-time | 6,312 (59·0%) | 19,151 (64·0%) | 1,445 (65·0%) | 690 (66·0%) | 1,264 (64·0%) |
| Part-time | 2,373 (22·0%) | 6,589 (22·0%) | 467 (21·0%) | 238 (23·0%) | 462 (23·0%) |
| Self-employed | 1,934 (18·0%) | 4,268 (14·0%) | 305 (14·0%) | 125 (12·0%) | 241 (13·0%) |
| **Employment Type at Recruitment in REACT** |  |  |  |  |  |
| Business and service | 596 (5·8%) | 1,494 (5·0%) | 96 (4·4%) | 51 (4·9%) | 123 (6·4%) |
| Care Home | 91 (0·9%) | 361 (1·2%) | 29 (1·3%) | 22 (2·1%) | 40 (2·1%) |
| Child-related | 756 (7·3%) | 32,912 (10·0%) | 253 (12·0%) | 131 (13·0%) | 241 (12·5%) |
| Health care worker | 967 (9·4%) | 3,348 (11·0%) | 355 (16·0%) | 172 (17·0%) | 317 (16·5%) |
| Logistic and security | 282 (2·7%) | 833 (2·8%) | 48 (2·2%) | 42 (4·0%) | 79 (4·1%) |
| Other essential worker/public facing | 1,513 (15·0%) | 4,227 (14·0%) | 342 (16·0%) | 140 (13·0%) | 310 (15·8%) |
| Work at home/not public facing | 6,115 (59·0%) | 16,134 (55·0%) | 1,049 (48·0%) | 480 (46·0%) | 815 (42·3%) |
| Missing | 299 | 729 | 45 | 15 | 42 |
| **Gender** |  |  |  |  |  |
| Female | 5,969 (56·0%) | 17,842 (59·0%) | 1,507 (68·0%) | 687 (65·0%) | 1,414 (72·0%) |
| Male | 4,650 (44·0%) | 12,165 (41·0%) | 710 (32·0%) | 366 (35·0%) | 553 (28·0%) |
| Missing | 0 | < 5 | 0 | 0 | 0 |
| **Age (years)** | 56 (47, 62) | 51 (41, 59) | 50 (42, 58) | 50 (41, 58) | 49 (41, 57) |
| **Ethnicity** |  |  |  |  |  |
| Asian | 354 (3·4%) | 858 (2·9%) | 75 (3·4%) | 37 (3·7%) | 51 (2·6%) |
| Black | 126 (1·2%) | 234 (0·8%) | 24 (1·1%) | 10 (1·0%) | 17 (0·9%) |
| Mixed | 115 (1·1%) | 440 (1·5%) | 44 (2·0%) | 21 (2·0%) | 30 (1·5%) |
| Other | 114 (1·1%) | 335 (1·1%) | 40 (1·8%) | 13 (1·3%) | 27 (1v4%) |
| White | 9,851 (93·0%) | 28,033 (94·0%) | 2,026 (92·0%) | 967 (92·0%) | 1,828 (94·0%) |
| Missing | 59 | 108 | 8 | 5 | 14 |
| **Education Level** |  |  |  |  |  |
| Degree level or higher | 4,828 (46·7%) | 15,822 (53·5%) | 1,159 (53·0%) | 558 (54·0%) | 917 (47·8%) |
| Other higher qualification below degree level | 1,139 (10·9%) | 2,979 (10·1%) | 219 (10·0%) | 113 (10·9%) | 213 (11·1%) |
| A-levels, NVQ levels 3 and equivalents | 1,694 (16·2%) | 4,739 (16·0%) | 384 (17·6%) | 170 (16·4%) | 377 (19·7%) |
| GCSE/O level | 1,442 (13·8%) | 3,322 (11·2%) | 246 (11·3%) | 108 (10·4%) | 238 (12·4%) |
| Qualification at level 1 and below | 504 (4·8%) | 1,036 (3·5%) | 64 (2·9%) | 31 (3·0%) | 73 (3·8%) |
| Other qualification | 551 (4·9%) | 1,210 (4·1%) | 81 (3·7%) | 41 (4·0%) | 57 (3·0%) |
| No qualification | 317 (3·0%) | 493 (1·7%) | 32 (1·5%) | 13 (1·3%) | 44 (2·3%) |
| Prefer not to say / Missing | 184 | 407 | 32 | 19 | 48 |
| **IMD Quintile** |  |  |  |  |  |
| Q1 – most deprived | 868 (8·9%) | 2,369 (8·5%) | 226 (11·0%) | 113 (11·0%) | 228 (12·0%) |
| Q2 | 1,544 (16·0%) | 4,363 (16·0%) | 338 (16·0%) | 168 (17·0%) | 324 (17·0%) |
| Q3 | 2,172 (22·0%) | 6,066 (22·0%) | 426 (21·0%) | 201 (20·0%) | 392 (21·0%) |
| Q4 | 2,452 (25·0%) | 6,976 (25·0%) | 499 (24·0%) | 229 (23·0%) | 455 (24·0%) |
| Q5 – least deprived | 2,688 (28·0%) | 8,037 (29·0%) | 585 (38·0%) | 279 (28·0%) | 466 (25·0%) |
| Missing | 895 | 2,197 | 143 | 63 | 102 |
| **If you were off work for two weeks due to illness how serious would the financial impact be on your household?** |  |  |  |  |  |
| Not serious at all | 2,247 (47·0%) | 6,567 (48·0%) | 470 (45·0%) | 252 (47·0%) | 374 (40·0%) |
| Not very serious | 1,422 (30·0%) | 4,194 (31·0%) | 326 (31·0%) | 141 (27·0%) | 276 (29·0%) |
| Fairly serious | 745 (16·0%) | 2,120 (15·0%) | 173 (17·0%) | 87 (16·0%) | 185 (20·0%) |
| Very serious | 356 (7·5%) | 852 (6·2%) | 73 (7·5%) | 51 (10·0%) | 109 (12·0%) |
| Missing | 5,849 | 16,275 | 1,175 | 522 | 1.023 |
| **Are you taking specific precautions because you are concerned that you will become severely ill with COVID-19?** |  |  |  |  |  |
| Yes | 1,113 (11·0%) | 2,185 (7·5%) | 225 (10·0%) | 110 (11·0%) | 250 (13·0%) |
| Missing | 299 | 729 | 45 | 15 | 42 |
| **Number of Comorbidities** |  |  |  |  |  |
| 0 | 6,221 (59·0%) | 18,358 (61·0%) | 1,191 (54·0%) | 562 (53·0%) | 903 (46.0%) |
| 1 | 2.801 (26·0%) | 7,647 (25·0%) | 622 (28·0%) | 286 (27·0%) | 562 (29.0%) |
| 2+ | 1,597 (15·0%) | 4,003 (13·0%) | 404 (18·0%) | 205 (19·0%) | 502 (26.0%) |
| **COVID-19 Symptoms Severity** |  |  |  |  |  |
| No symptoms | - | 1,547 (5·0%) | 0 (0·0%) | 0 (0·0%) | 0 (0·0%) |
| Mild symptoms | - | 7,613 (25·0%) | 216 (10·0%) | 234 (12·0%) | 146 (14·0%) |
| Moderate symptoms | - | 15,792 (53·0%) | 938 (42·0%) | 849 (43·0%) | 455 (43·0%) |
| Severe symptoms | - | 5,056 (17·0%) | 1,063 (48·0%) | 884 (45·0%) | 452 (43·0%) |

**Supplementary Table 3. Associations between Long COVID status with being in paid work and changes in hours of work at follow-up according to participants’ gender.**

|  | **In Paid Work at time of Recruitment in the Follow-up Survey** | |  | **Change in Hours of Paid Work** | |
| --- | --- | --- | --- | --- | --- |
|  | Male | Female |  | Male | Female |
| No COVID | 0·98  (0·89, 1·09) | 0·97  (0·90, 1·06) |  | 0·92  (0·80, 1·04) | 1·01  (0·91, 1·12) |
| Asymptomatic or resolved short COVID-19 < 4 weeks | Reference | Reference |  | Reference | Reference |
| Resolved short COVID-19 > 4 to < 12 weeks | 0·91  (0·72, 1·16) | 0·96  (0·82, 1·13) |  | 2·25  (1·81, 2·80) | 1·73  (1·49, 2·00) |
| Resolved Long COVID | 0·75  (0·55, 1·03) | 0·83  (0.67, 1.04) |  | 1·99  (1·47, 2·69) | 2·00  (1·61, 2·47) |
| Unresolved Long COVID | 0·60  (0·47, 0·77) | 0·63  (0.54, 0.73) |  | 5·04  (4·06, 6·25) | 4·18  (3·66, 4·75) |
| *p-value for the interaction term* | *0.97* | |  | *0*·*08* | |

Adjusted odds ratio (aOR) and 95% confidence intervals (CI). Models were adjusted for nature of employment at time of recruitment in the REACT study, type of employment at time of recruitment in the REACT study, gender, age, previous comorbidities, IMD (index of multiple deprivation) quintiles, ethnicity, self-reported severity of COVID-19 symptoms, taking precautions at time of recruitment in the REACT study due to worries about becoming ill (yes/no), financial impact in case of having to take two weeks off work due to illness, and the interaction between gender and Long COVID status. When the outcome was whether a participant was in paid work at time in the follow-up survey, we additionally adjusted for months between recruitment in the REACT study and recruitment in the follow-up survey. The p-value for the interaction term is derived from a likelihood ratio test comparing models with and without the interaction.

**Supplementary Table 4. Associations between Long COVID status and clusters of persistent symptoms with being in paid work and changes in hours of work at follow-up according to participants’ socio-economic status measured by IMD quintiles.**

|  | **In Paid work at time of Recruitment in the Follow-up Survey** | | | | |  | **Changes of Hours of Paid Work** | | | | |
| --- | --- | --- | --- | --- | --- | --- | --- | --- | --- | --- | --- |
|  | Q1 – most deprived | Q2 | Q3 | Q4 | Q5 – least deprived |  | Q1 – most deprived | Q2 | Q3 | Q4 | Q5 – least deprived |
| No COVID | 0·92  (0·73, 1·16) | 1·01  (0·84, 1·21) | 0·93  (0·69, 1·24) | 0·98  (0·86, 1·12) | 0·98  (0·86, 1·12) |  | 1·02  (0·79, 1·30) | 0·90  (0·74, 1·10) | 0·92  (0·77, 1·09) | 0.81  (0·62, 0·97) | 1·93  (1·45, 2·56) |
| Asymptomatic or resolved short COVID-19 < 4 weeks | Reference | Reference | Reference | Reference | Reference |  | Reference | Reference | Reference | Reference | Reference |
| Resolved short COVID-19 > 4 to < 12 weeks | 0·88  (0·58, 1·34) | 1·08  (0·76, 1·56) | 0·93  (0·69, 1·24) | 0·82  (0·63, 1·07) | 1·05  (0·81, 1·36) |  | 1·94  (1·37, 2·76) | 1·52  (1·12, 2·07) | 1·73  (1·33, 2·25) | 2·42  (1·90, 3·07) | 1·77  (1·38, 2·26) |
| Resolved Long COVID | 0·91  (0·50, 1·63) | 0·67  (0·44, 1·04) | 0·88  (0·57, 1·37) | 0·81  (0·55, 1·19) | 0·81  (0·57, 1·13) |  | 1.74  (1.06, 2.86) | 1·71  (1·11, 2·62) | 2·20  (1·53, 3·18) | 2·13  (1·50, 3·04) | 2·06  (1·46, 2·92) |
| Unresolved Long COVID | 0·59  (0·41, 0·87) | 0·66  (0·49, 0·90) | 0·55  (0·42, 0·72) | 0·63  (0·48, 0·81) | 0·67  (0·52, 0·86) |  | 3·16  (2·27, 4·41) | 4·17  (3·20, 4·44) | 4·29  (3·36, 5·48) | 4·70  (3·72, 5·94) | 5·18  (4·15, 6·47) |
| *p-value for the interaction term* | *0*·*99* | | | | |  | *0*·*30* | | | | |

Adjusted odds ratio (aOR) and 95% confidence intervals (CI). Models were adjusted for nature of employment at time of recruitment in the REACT study, type of employment at time of recruitment in the REACT study, gender, age, previous comorbidities, IMD (index of multiple deprivation) quintiles, ethnicity, education level, self-reported severity of COVID-19 symptoms, taking precautions at time of recruitment in the REACT study due to worries about becoming ill (yes/no) , financial impact in case of having to take two weeks off work due to illness, and the interaction between gender and Long COVID status. When the outcome was whether a participant was in paid work at time in the follow-up survey, we additionally adjusted for months between recruitment in the REACT study and recruitment in the follow-up survey. The p-value for the interaction term is derived from a likelihood ratio test comparing models with and without the interaction.

**Supplementary Table 5. Participants characteristics according to cluster membership for the three-cluster solution. Clusters were derived using a hierarchical clustering algorithm. Cluster 1 (N = 1,048) describes a fatigue-predominant Long COVID. Cluster 2 (N = 466) describes Long COVID characterised mainly by loss/change of smell and taste. Cluster 3 (N = 694) describes multisystem severe Long COVID**

|  | Cluster 1  (N = 1,048) | Cluster 2  (N = 466) | Cluster 3  (N = 694) | Overall  (N = 2,208) |  |  |
| --- | --- | --- | --- | --- | --- | --- |
| **Employment Nature at Recruitment in REACT** |  |  |  |  |  |  |
| Full-time | 678 (64·7%) | 284 (60·9%) | 443 (63·8%) | 1,405 (63·6%) |  |  |
| Part-time | 233 (22·2%) | 126 (27·1%) | 167 (24·1%) | 526 (23·8%) |  |  |
| Self-employed | 137 (13·1%) | 56 (12·0%) | 84 (12·1%) | 277 (12·6%) |  |  |
| **Employment Type at Recruitment in REACT** |  |  |  |  |  |  |
| Business and service | 54 (5·3%) | 26 (5·7%) | 43 (6·3%) | 123 (5·7%) |  |  |
| Care Home | 16 (1·6%) | 9 (2·0%) | 19 (2·7%) | 44 (2·0%) |  |  |
| Child-related | 135 (13·%) | 65 (14·2%) | 80 (11·7%) | 280 (12·9%) |  |  |
| Health care worker | 172 (16·7%) | 75 (16·4%) | 80 (11·7%) | 382 (17·6%) |  |  |
| Logistic and security | 45 (4·4%) | 15 (3·3%) | 32 (4·7%) | 92 (4·2%) |  |  |
| Other essential worker/public facing | 160 (15·6%) | 57 (12·5%) | 116 (17·0%) | 333 (15·4%) |  |  |
| Work at home/not public facing | 446 (43·4%) | 210 (46·0%) | 259 (37·9%) | 915 (41·2%) |  |  |
| Missing | 20 | 9 | 10 | 39 |  |  |
| **Gender** |  |  |  |  |  |  |
| Female | 725 (69·2%) | 343 (73·6%) | 524 (75·5%) | 1,592 (72·1%) |  |  |
| Male | 323 (30·8%) | 123 (26·4%) | 170 (24·5%) | 616 (27·9%) |  |  |
| **Age (years)** | 49 (41, 57) | 50 (41, 57) | 51 (43, 57) | 50 (42, 57) |  |  |
| **Ethnicity** |  |  |  |  |  |  |
| Asian | 31 (3·0%) | 6 (1·3%) | 16 (2·3%) | 53 (2·4%) |  |  |
| Black | 7 (0·7%) | <5 (0·4%) | 8 (1·2%) | 17 (0·8%) |  |  |
| Mixed | 15 (1·3%) | 9 (2·0%) | 11 (1·6%) | 35 (1·6%) |  |  |
| Other | 11 (1·1%) | 6 (1·3%) | 11 (1·6%) | 28 (1·4%) |  |  |
| White | 981 (93·9%) | 438 (95·0%) | 641 (93·3%) | 2,060 (93·9%) |  |  |
| Missing | <5 | 5 | 7 | 15 |  |  |
| **Education Level** |  |  |  |  |  |  |
| Degree level or higher | 581 (56·4%) | 242 (52·4%) | 286 (42·3%) | 1,109 (51·1%) |  |  |
| Other higher qualification below degree level | 107 (10·4%) | 39 (8·4%) | 83 (12·3%) | 220 (10·6%) |  |  |
| A-levels, NVQ levels 3 and equivalents | 179 (17·4%) | 78 (16·9%) | 149 (2·0%) | 406 (18·7%) |  |  |
| GCSE/O level | 93 (9·0%) | 63 (13·6%) | 85 (12·6%) | 241 (11·1%) |  |  |
| Qualification at level 1 and below | 25 (2·5%) | 17 (3·7%) | 25 (3·7%) | 68 (3·1%) |  |  |
| Other qualification | 28 (2·7%) | 13 (2·8%) | 24 (5·0%) | 75 (3·5%) |  |  |
| No qualification | 16 (1·6%) | 10 (2·2%) | 15 (2·2%) | 41 (1·9%) |  |  |
| Prefer not to say / Missing | 18 | < 5 | 17 | 39 |  |  |
| **IMD Quintile** |  |  |  |  |  |  |
| Q1 – most deprived | 119 (11·9%) | 51 (11·5%) | 88 (13·2%) | 258 (12·2%) |  |  |
| Q2 | 165 (16·6%) | 74 (16·7%) | 124 (18·6%) | 363 (17·2%) |  |  |
| Q3 | 193 (19·4%) | 96 (21·6%) | 158 (23·7%) | 447 (21·2%) |  |  |
| Q4 | 238 (28·3%) | 104 (23·4%) | 155 (23·3%) | 497 (23·6%) |  |  |
| Q5 – least deprived | 282 (28·3%) | 119 (26·8%) | 141 (21·7%) | 542 (25·7%) |  |  |
| Missing | 51 | 22 | 28 | 101 |  |  |
| **If you were off work for two weeks due to illness how serious would the financial impact be on your household?** |  |  |  |  |  |  |
| Not serious at all | 231 (45·7%) | 122 (50·6%) | 122 (33·9%) | 475 (42·9%) |  |  |
| Not very serious | 140 (27·7%) | 56 (23·2%) | 109 (30·3%) | 305 (27·6%) |  |  |
| Fairly serious | 86 (17·0%) | 44 (18·3%) | 76 (21·1%) | 206 (18·6%) |  |  |
| Very serious | 49 (9·7%) | 19 (7·9%) | 53 (14·7%) | 121 (10·9%) |  |  |
| Missing | 542 | 225 | 334 | 1,101 |  |  |
| **Are you taking specific precautions because you are concerned that you will become severely ill with COVID-19?** |  |  |  |  |  |  |
| Yes | 117 (11·4%) | 39 (8·6%) | 141 (20·6%) | 297 (13·7%) |  |  |
| Missing | 20 | 9 | 10 | 39 |  |  |
| **COVID-19 infection severity** |  |  |  |  |  |  |
| Mild Symptoms | 91 (8·7%) | 666 (14·2%) | 26 (3·8%) | 183 (8·3%) |  |  |
| Moderate Symptoms | 459 (43·8%) | 220 (47·2%) | 229 (33·0%) | 908 (41·1%) |  |  |
| Severe Symptoms | 498 (47·5%) | 180 (38·6%) | 439 (63·3%) | 1,117 (50·6%) |  |  |
| **Number of comorbidities** |  |  |  |  |  |  |
| 0 | 512 (48·9%) | 264 (56·7%) | 240 (34·5%) | 1.016 (46·0%) |  |  |
| 1 | 316 (30·2%) | 112 (24·0%) | 201 (29·0%) | 629 (28·5%) |  |  |
| 2+ | 220 (21·0%) | 90 (19·3%) | 253 (36·5%) | 563 (25·5%) |  |  |
| **In paid work at time of recruitment in REACT-LC** | 926 (88·4%) | 417 (89·5%) | 566 (81·6%) | 1,909 (86·5%) |  |  |
| **Has your physical/mental health affected the number of hours of paid work that you can do?** |  |  |  |  |  |  |
| Yes | 282 (28·6%) | 86 (19·6%) | 361 (57·1%) | 729 (35·5%) |  | 739 (35·5%) |
| Missing | 62 | 28 | 62 | 152 |  |  |

**Supplementary Table 6. Participants characteristics according to cluster membership for the two-cluster solution. Clusters were derived using a hierarchical clustering algorithm. Cluster 1 includes participants with fatigue-predominant Long COVID while cluster 2 includes participants with multisystem severe Long COVID.**

|  | Cluster 1  (N = 1,514) | Cluster 2  (N = 694) | Overall  (N = 2,208) |
| --- | --- | --- | --- |
| **Employment Nature at Recruitment in REACT** |  |  |  |
| Full-time | 962 (63·5%) | 443 (63·8%) | 1,405 (63·6%) |
| Part-time | 359 (23·7%) | 167 (24·1%) | 526 (23·8%) |
| Self-employed | 193 (12·6%) | 84 (12·1%) | 277 (12·6%) |
| **Employment Type at Recruitment in REACT** |  |  |  |
| Business and service | 80 (5·4%) | 43 (6·3%) | 123 (5·7%) |
| Care Home | 25 (1·7%) | 19 (2·8%) | 44 (2·0%) |
| Child-related | 200 (13·5%) | 80 (5·4%) | 280 (12·9%) |
| Health care worker | 247 (16·6%) | 135 (19·7%) | 382 (17·6%) |
| Logistic and security | 60 (4·0%) | 32 (4·7%) | 92 (4·2%) |
| Other essential worker/public facing | 217 (14·6%) | 116 (17·0%) | 333 (15·4%) |
| Work at home/not public facing | 656 (44·2%) | 259 (37·9%) | 915 (41·2%) |
| Missing | 29 | 10 | 39 |
| **Gender** |  |  |  |
| Female | 1,068 (70·5%) | 524 (75·5%) | 1,592 (72·1%) |
| Male | 446 (29·5%) | 170 (24·5%) | 616 (27·9%) |
| **Age (years)** | 49 (41, 57) | 51 (43, 57) | 50 (42, 57) |
| **Ethnicity** |  |  |  |
| Asian | 37 (2·5%) | 16 (2·3%) | 53 (2·4%) |
| Black | 9 (0·6%) | 8 (1·2%) | 17 (0·8%) |
| Mixed | 24 (1·6%) | 11 (1·6%) | 35 (1·6%) |
| Other | 17 (1·1%) | 11 (1·6%) | 28 (1·4%) |
| White | 1,419 (94·2%) | 641 (93·3%) | 2,060 (93·9%) |
| Missing | 8 | 7 | 15 |
| **Education Level** |  |  |  |
| Degree level or higher | 823 (55·2%) | 286 (42·3%) | 1,109 (51·1%) |
| Other higher qualification below degree level | 146 (9·8%) | 83 (12·3%) | 220 (10·6%) |
| A-levels, NVQ levels 3 and equivalents | 257 (11·2%) | 149 (22·0%) | 406 (18·7%) |
| GCSE/O level | 156 (10·5%) | 85 (12·6%) | 241 (11·1%) |
| Qualification at level 1 and below | 43 (2·9%) | 25 (3·7%) | 68 (3·1%) |
| Other qualification | 41 (2·8%) | 34 (5·0%) | 75 (3·5%) |
| No qualification | 26 (1·7%) | 15 (2·2%) | 41 (1·9%) |
| Prefer not to say / Missing | 22 | 17 | 39 |
| **IMD Quintile** |  |  |  |
| Q1 – most deprived | 170 (11·8%) | 88 (13·2%) | 258 (12·2%) |
| Q2 | 239 (16·6%) | 124 (18·6%) | 363 (17·2%) |
| Q3 | 289 (20·1%) | 158 (23·7%) | 447 (21·2%) |
| Q4 | 342 (23·7%) | 155 (23·3%) | 497 (23·6%) |
| Q5 – least deprived | 401 (27·8%) | 141 (22·2%) | 542 (25·7%) |
| Missing | 73 | 28 | 101 |
| **If you were off work for two weeks due to illness how serious would the financial impact be on your household?** |  |  |  |
| Not serious at all | 353 (47·3%) | 122 (33·9%) | 475 (42·9%) |
| Not very serious | 196 (26·2%) | 109 (30·3%) | 305 (37·6%) |
| Fairly serious | 130 (17·4%) | 76 (21·1%) | 206 (18·6%) |
| Very serious | 68 (9·1%) | 53 (14·7%) | 121 (10·9%) |
| Missing | 767 | 334 | 1,101 |
| **Are you taking specific precautions because you are concerned that you will become severely ill with COVID-19?** |  |  |  |
| Yes | 156 (10·5%) | 141 (20·6%) | 297 (13·7%) |
| Missing | 29 | 10 | 39 |
| **COVID-19 infection severity** |  |  |  |
| Mild Symptoms | 157 (10·4%) | 26 (3·8%) | 183 (8·3%) |
| Moderate Symptoms | 679 (44·9%) | 229 (33·0%) | 908 (41·1%) |
| Severe Symptoms | 678 (44·8%) | 439 (63·3%) | 1,117 (50·6%) |
| **Number of comorbidities** |  |  |  |
| 0 | 776 (51·3%) | 240 (34·6%) | 1.016 (46·0%) |
| 1 | 428 (28·3%) | 201 (28·9%) | 629 (28·5%) |
| 2+ | 310 (20·5%) | 253 (36·5%) | 563 (25·5%) |
| **In paid work at time of recruitment in REACT-LC** | 1,343 (88·7%) | 566 (81·6%) | 1,909 (86·5%) |
| **Has your physical/mental health affected the number of hours of paid work that you can do?** |  |  |  |
| Yes | 368 (25·8%) | 361 (57·1%) | 729 (35·5%) |
| Missing | 90 | 62 | 152 |

**Supplementary Table 7. Associations between Long COVID symptoms clusters with being in paid work and changes in hours of work at follow-up in the fully-adjusted model. Symptoms clusters were derived using hierarchical clustering with two cluster solutions**

|  | In Paid Work at Time of Recruitment in the Follow-Up Survey | Changes in Hours of Paid Work |  |
| --- | --- | --- | --- |
|  | aOR (95% CI) | aOR (95% CI) |  |
| **Long COVID Symptoms Clusters** |  |  |  |
| C1: Fatigue-Predominant Long COVID | Reference | Reference |  |
| C2: Multisystem severe Long COVID | 0·59 (0·44, 0·78) | 3·28 (2·66, 4·07) |  |

Adjusted odds ratio (aOR) and 95% confidence intervals (CI). Models were adjusted for nature of employment at time of recruitment in the REACT study, type of employment at time of recruitment in the REACT study, gender, age, previous comorbidities, IMD (index of multiple deprivation) quintiles, ethnicity, education level, self-reported severity of COVID-19 symptoms, taking precautions at time of recruitment in the REACT study due to worries about becoming ill (yes/no), financial impact in case of having to take two weeks off work due to illness, and the interaction between gender and Long COVID status. When the outcome was whether a participant was in paid work at time in the follow-up survey, we additionally adjusted for months between recruitment in the REACT study and recruitment in the follow-up survey.

**Supplementary Table 8. Frequency and prevalence of Long COVID status among participants with confirmed and suspected infections.**

| **Long COVID Status** | **N (%)** |
| --- | --- |
| No COVID-19 | 10,619 (21·5%) |
| Asymptomatic or resolved short COVID-19 less than 4 weeks | 31,622 (64·2%) |
| Resolved COVID-19 (4 – 11 weeks) | 3,130 (6·4%) |
| Resolved Long COVID | 2,341 (3·1%) |
| Unresolved Long COVID | 1,521 (4·8%) |

**Supplementary Table 9. Associations between Long COVID status with being in paid work and changes in hours of work at follow-up. Long COVID status was computed including both confirmed by test and suspected infections.**

|  | In Paid Work at Time of Recruitment in the Follow-Up Survey | Changes in Hours of Paid Work |  |
| --- | --- | --- | --- |
|  | aOR (95% CI) | aOR (95% CI) |  |
| **Model #1 Exposure: Long COVID Status** |  |  |  |
| No COVID | 1·00 (0·94, 1·06) | 0·97 (0·90, 1·05) |  |
| Asymptomatic or resolved short COVID-19 < 4 weeks | Reference | Reference |  |
| Resolved COVID-19 (4 – 11 weeks) | 0·93 (0.83, 1·04) | 1·87 (1·68, 2·08) |  |
| Resolved Long COVID | 0·77 (0.67, 0·89) | 2·18 (1·89, 2·52) |  |
| Unresolved Long COVID | 0·65 (0.58, 0·73) | 4·50 (4·05, 5·01) |  |

Adjusted odds ratio (aOR) and 95% confidence intervals (CI). Models were adjusted for nature of employment at time of recruitment in the REACT study, type of employment at time of recruitment in the REACT study, gender, age, education level, previous comorbidities, IMD (index of multiple deprivation) quintiles, ethnicity, self-reported severity of COVID-19 symptoms, taking precautions at time of recruitment in the REACT study due to worries about becoming ill (yes/no), and financial impact in case of having to take two weeks off work due to illness. When the outcome was whether a participant was in paid work at time in the follow-up survey, we additionally adjusted for months between recruitment in the REACT study and recruitment in the follow-up survey.

**Supplementary Table 10.Characteristics of participants included in the sensitivity analyses**

|  | Sensitivity Analysis #1  (N = 83,468) | Sensitivity Analysis #2  (N = 36,458) |
| --- | --- | --- |
| **Employment Nature at Recruitment in REACT** | |  |
| Full-time | 28,862 (35·5%) | 22,713 (62·3%) |
| Part-time | 10,129 (12·4%) | 8,141 (22·3%) |
| Retired | 28,925 (35·5%) | - |
| Self-employed | 6,873 (8·4%) | 5,604 (15·4%) |
| Sick/disabled | 1,172 (1·4%) | - |
| Student | 1,381 (1·7%) | - |
| Not working | 4,060 (5·0%) | - |
| Missing | 2,066 | - |
| **Employment Type at Recruitment in REACT**** | |  |
| Business and service | 2,330 (2·9%) | 1,803 (5·1%) |
| Care Home | 543 (0·7%) | 365 (1·0%) |
| Child-related | 4,293 (5·4%) | 3,154 (8·9%) |
| Health care worker | 5,159 (6·4%) | 3,688 (10·4%) |
| Logistic and security | 1,284 (1·6%) | 957 (2·7%) |
| Not in full-time, part-time nor self-employed | 35.538 (44·1%) | - |
| Other essential worker | 26,532 (8·1%) | 5,247 (14·8%) |
| Work at home/not public facing | 24,593 (30·6%) | 20,199 (57·0%) |
| Missing | 3,196 | 1,045 |
| **Gender** |  |  |
| Male | 34,431 (41·3%) | 14,942 (41·0%) |
| Female | 49,035 (58·9%) | 21,515 (59·0%) |
| Missing | < 5 | < 5 |
| **Age (years)** | 60 (48, 69) | 53 (43, 60) |
| **Long COVID Status** |  |  |
| No COVID-19 | 26,360 (31·6%) | 10,619 (29·1%) |
| Asymptomatic or resolved short COVID-19 less than 4 weeks | 49,567 (59·4%) | 23,133 (63·5%) |
| Resolved short COVID-19 >4 to <12 weeks: | 3,293 (4·0%) | 1,268 (3·5%) |
| Resolved Long COVID | 1,446 (1·73%) | 452 (1·2%) |
| Unresolved Long COVID | 2,802 (3·36%) | 986 (2·7%) |
| **Ethnicity** |  |  |
| Asian | 2,098 (2·5%) | 1,066 (2·9%) |
| Black | 570 (2·5%) | 316 (0·9%) |
| Mixed | 901 (1·1%) | 495 (1·4%) |
| Other | 869 (1·1%) | 395 (1·1%) |
| White | 78,410 (94·6%) | 34,044 (93·7%) |
| Missing | 620 | 182 |
| **Education Level** |  |  |
| Degree level or higher | 37,090 (45·5%) | 18,429 (51·3%) |
| Other higher qualification below degree level | 9,413 (11·6%) | 3,741 (10·4%) |
| A-levels, NVQ levels 3 and equivalents | 11,896 (14·6% | 5,792 (16·1%) |
| GCSE/O level | 10,400 (12·8%) | 4,326 (12·0%) |
| Qualification at level 1 and below | 3,433 (4·21%) | 1,383 (3·9%) |
| Other qualification | 5,520 (6·8%) | 1,529 (4·3%) |
| No qualification | 3,735 (4·6%) | 723 (2·0%) |
| Prefer not to say / Missing | 1,981 | 535 |
| **IMD Quintile** |  |  |
| Q1 – most deprived | 6,157 (8·0%) | 2,818 (8·5%) |
| Q2 | 11,344 (14·7%) | 5,183 (15·5%) |
| Q3 | 16,435 (21·3%) | 7,333 (22·0%) |
| Q4 | 19,713 (25·6%) | 8,368 (25·1%) |
| Q5 – least deprived | 23,430 (30·4%) | 9,643 (29·0%) |
| Missing | 6,389 | 3,113 |
| **If you were off work for two weeks due to illness how serious would the financial impact be on your household?** | |  |
| Not serious at all | 9,911 (47·2%) | 7,385 (48·4%) |
| Not very serious | 6,359 (30·3%) | 4,626 (30·3%) |
| Fairly serious | 3,310 (15·8%) | 2,298 (15·1%) |
| Very serious | 1,441 (6·9%) | 954 (6·3%) |
| Missing | 62,447 | 21,195 |
| **Are you taking specific precautions because you are concerned that you will become severely ill with COVID-19?** |  |  |
| Yes | 11,289 (13·9%) | 3,050 (8·6%) |
| Missing | 2,036 | 1,044 |
| **Number of Comorbidities***** |  |  |
| 0 | 45,204 (54·2%) | 21,914 (60·1%) |
| 1 | 23,345 (28·0%) | 9,366 (25·7%) |
| 2+ | 14,919 (17·8%) | 5,178 (14·2%) |
| **In paid work at time of recruitment in the REACT 2022 follow-up survey** | 42,177 (50·5%) | 31,150 (85·4%) |
| **Has you physical/mental health affected the number of hours of paid work that you can do?** |  |  |
| Yes | 6,654 (8·7%) | 3,415 (9·8%) |
| Missing | 7,333 | 1,625 |

Notes: sensitivity analysis #1 includes all participants in the REACT 2022 follow-up regardless of their employment status at recruitment in the REACT study, Sensitivity analysis #2 includes participants with known COVID-19 history who were in paid work and had not yet had an infection at recruitment in REACT.

**Supplementary Table 11.Associations between Long COVID status and symptoms with being in paid work and changes in hours of work at follow-up. All participants, regardless of their baseline employment, in the REACT-LC 2022 follow-up survey with known history of COVID-19 are included (N = 83,648).**

|  | In Paid Work at Time of Recruitment in the Follow-Up Survey | Changes in Hours of Paid Work |  |
| --- | --- | --- | --- |
|  | aOR (95% CI) | aOR (95% CI) |  |
| **Model #1 Exposure: Long COVID Status** |  |  |  |
| No COVID | 0·88 (0·84, 0·93) | 1·01 (0·95, 1·08) |  |
| Asymptomatic or resolved short COVID-19 < 4 weeks | Reference | Reference |  |
| Resolved COVID-19 (4 – 11 weeks) | 0·98 (0·88, 1·11) | 1·79 (1·59, 2·02) |  |
| Resolved Long COVID | 0·82 (0·70, 0·96) | 1·86 (1·59, 2·18) |  |
| Unresolved Long COVID | 0·68 (0·61, 0·76) | 4·37 (3·94, 4·85) |  |
| **Model #2 Exposure: Long COVID Symptoms Clusters** |  |  |  |
| C1: Fatigue-Predominant Long COVID | Reference | Reference |  |
| C2: Long COVID characterised by loss/change of smell and/or taste | 1·18 (0·92, 1·53) | 0·38 (0·31, 0·47) |  |
| C3: Multisystem severe Long COVID | 0·69 (0·50, 0·94) | 2·16 (1·69, 2·76) |  |

Adjusted odds ratio (aOR) and 95% confidence intervals (CI). Models were adjusted for nature of employment at time of recruitment in the REACT study, type of employment at time of recruitment in the REACT study, gender, age, previous comorbidities, IMD (index of multiple deprivation) quintiles, ethnicity, education level, self-reported severity of COVID-19 symptoms, taking precautions at time of recruitment in the REACT study due to worries about becoming ill (yes/no), financial impact in case of having to take two weeks off work due to illness, and the interaction between gender and Long COVID status. When the outcome was whether a participant was in paid work at time in the follow-up survey, we additionally adjusted for months between recruitment in the REACT study and recruitment in the follow-up survey.

**Supplementary Table 12**.**Associations between Long COVID status and symptoms with being in paid work and changes in hours of work at follow-up. Participants are included if they were in paid employment at baseline and had yet to develop their first COVID-19 infection (N = 36,458).**

|  | In Paid Work at Time of Recruitment in the Follow-Up Survey | Changes in Hours of Paid Work |  |
| --- | --- | --- | --- |
|  | aOR (95% CI) | aOR (95% CI) |  |
| **Model #1 Exposure: Long COVID Status** |  |  |  |
| No COVID | 0·97 (0·90, 1·04) | 1·10 (1·00, 1·21) |  |
| Asymptomatic or resolved short COVID-19 < 4 weeks | Reference | Reference |  |
| Resolved short COVID-19 (4 – 11 weeks) | 0·91 (0·77, 1·07) | 2·01 (1·71, 2·38) |  |
| Resolved Long COVID | 0·93 (0·70, 1·23) | 2·46 (1·89, 3·18) |  |
| Unresolved Long COVID | 0·65 (0·55, 0·78) | 4·38 (3·75, 5·12) |  |
| **Model #2 Exposure: Long COVID Symptoms Clusters (N = 933)** |  |  |  |
| C1: Fatigue-Predominant Long COVID | Reference | Reference |  |
| C2: Long COVID characterised by loss/change of smell and/or taste | 1·18 (0·70, 2·01) | 0·35 (0·24, 0·51) |  |
| C3: Multisystem severe Long COVID | 0·66 (0·38, 1·14) | 1·75 (1·21, 2·52) |  |

Adjusted odds ratio (aOR) and 95% confidence intervals (CI). Models were adjusted for nature of employment at time of recruitment in the REACT study, type of employment at time of recruitment in the REACT study, gender, age, previous comorbidities, IMD (index of multiple deprivation) quintiles, ethnicity, education level, self-reported severity of COVID-19 symptoms, taking precautions at time of recruitment in the REACT study due to worries about becoming ill (yes/no), financial impact in case of having to take two weeks off work due to illness, and the interaction between gender and Long COVID status. When the outcome was whether a participant was in paid work at time in the follow-up survey, we additionally adjusted for months between recruitment in the REACT study and recruitment in the follow-up survey.

**Supplementary Table 13. Associations between Long COVID symptoms clusters with being in paid work and changes in hours of work at follow-up. Symptoms clusters were derived with the PAM algorithm**

|  | In Paid Work at Time of Recruitment in the Follow-Up Survey | Changes in Hours of Paid Work |  |
| --- | --- | --- | --- |
|  | aOR (95% CI) | aOR (95% CI) |  |
| **Long COVID Symptoms Clusters** |  |  |  |
| C1: Fatigue-Predominant Long COVID | Reference | Reference |  |
| C2: Long COVID characterised by loss/change of smell and/or taste | 1·33 (0·93, 1·90) | 0·69 (0·53, 0·90) |  |
| C3: Multisystem severe Long COVID | 0·64 (0·47, 0·86) | 2·96 (2·34, 3·74) |  |

Adjusted odds ratio (aOR) and 95% confidence intervals (CI). Models were adjusted for nature of employment at time of recruitment in the REACT study, type of employment at time of recruitment in the REACT study, gender, age, previous comorbidities, IMD (index of multiple deprivation) quintiles, ethnicity, education level, self-reported severity of COVID-19 symptoms, taking precautions at time of recruitment in the REACT study due to worries about becoming ill (yes/no), financial impact in case of having to take two weeks off work due to illness, and the interaction between gender and Long COVID status. When the outcome was whether a participant was in paid work at time in the follow-up survey, we additionally adjusted for months between recruitment in the REACT study and recruitment in the follow-up survey.

**REFERENCE**

1 Elliott P, Whitaker M, Tang D, *et al.* Design and Implementation of a National SARS-CoV-2 Monitoring Program in England: REACT-1 Study. *Am J Public Health* 2023; **113**: 545–54.

2 Ward H, Atchison C, Whitaker M, *et al.* Design and Implementation of a National Program to Monitor the Prevalence of SARS-CoV-2 IgG Antibodies in England Using Self-Testing: The REACT-2 Study. *Am J Public Health* 2023; **113**: 1201–9.

3 Atchison C, Davies B, Cooper E, *et al.* Long-term health impacts of COVID-19 among 242,712 adults in England. *Nat Commun* 2023; **14**. DOI:https://doi.org/10.1038/s41467-023-41879-2.

4 Van Buuren S, Groothuis-Oudshoorn K. mice: Multivariate Imputation by Chained Equations in R. *J Stat Softw*; **45**: 1–67.

5 Bodinier B, Rodrigues S, Karimi M, Filippi S, Chiquet J, Chadeau-Hyam M. Stability Selection and Consensus Clustering in R: The R Package sharp. *J Stat Softw* 2025; **112**: 1–27.

6 Kaufman L, Rousseeuw PJ. Finding groups in data: an introduction to cluster analysis. John Wiley & Sons, Inc., 1990.

7 Office for National Statistics. Lower layer Super Output Area population estimates (Accredited official statistics). 2025. https://www.ons.gov.uk/peoplepopulationandcommunity/populationandmigration/populationestimates/datasets/lowersuperoutputareamidyearpopulationestimatesnationalstatistics (accessed April 20, 2026).

8 Office for National Statistics. Prevalence of ongoing symptoms following coronavirus (COVID-19) infection in the UK. 2023. https://www.ons.gov.uk/peoplepopulationandcommunity/healthandsocialcare/conditionsanddiseases/datasets/alldatarelatingtoprevalenceofongoingsymptomsfollowingcoronaviruscovid19infectionintheuk (accessed April 20, 2026).

9 Office for National Statistics. Ethnic Group by Age and Sex in england and Wales. https://www.ons.gov.uk/peoplepopulationandcommunity/culturalidentity/ethnicity/datasets/ethnicgroupbyageandsexinenglandandwales (accessed April 20, 2026).
